# Targeting treatment of bladder cancer using PTK7 aptamer-gemcitabine conjugate

**DOI:** 10.1186/s40824-022-00328-9

**Published:** 2022-12-05

**Authors:** Wei Xiang, Yongbo Peng, Hongliang Zeng, Chunping Yu, Qun Zhang, Biao Liu, Jiahao Liu, Xing Hu, Wensu Wei, Minhua Deng, Ning Wang, Xuewen Liu, Jianfei Xie, Weibin Hou, Jin Tang, Zhi Long, Long Wang, Jianye Liu

**Affiliations:** 1 Department of Urology, The Third Xiangya Hospital of Central South University, No.138, Tongzipo Road, Changsha, Hunan 410013 China; 2 Chongqing Key Laboratory for Pharmaceutical Metabolism Research, the Key Laboratory of Biochemistry and Molecular Pharmacology, College of Pharmacy, Chongqing Medical University, No.1, Yixueyuan Road, Chongqing, 400016 China; 3 Institute of Chinese Materia Medica, Hunan Academy of Chinese Medicine, No.8, Yuehua Road, Changsha, 410013 China; 4 Department of Urology, Sun Yat-sen University Cancer Center, No. 651, Dongfeng Road East, Guangzhou, Guangdong 510060 China; 5 State Key Laboratory of Oncology in Southern China, Collaborative Innovation Center for Cancer Medicine, No. 651, Dongfeng Road East, Guangzhou, Guangdong 510060 China; 6 Department of Radiotherapy, The First Affiliated Hospital of Sun Yat-sen University, 58 Zhongshan 2nd Road, Guangzhou, Guangdong 510080 China; 7 Department of Onology, The Third Xiangya Hospital of Central South University, No.138, Tongzipo Road, Changsha, Hunan 410013 China; 8 Department of Nursing, The Third Xiangya Hospital of Central South University, No.138, Tongzipo Road, Changsha, Hunan 410013 China

**Keywords:** Aptamer, PTK7, Bladder cancer, Targeted therapy, PTK7-GEMs

## Abstract

**Background:**

Gemcitabine (GEM) is one of the first-line chemotherapies for bladder cancer (BC), but the GEMs cannot recognize cancer cells and have a low long-term response rate and high recurrence rate with side effects during the treatment of BC. Targeted transport of GEMs to mediate cytotoxicity to tumor and avoid the systemic side effects remains a challenge in the treatment of BC.

**Methods:**

Based on a firstly confirmed biomarker in BC-protein tyrosine kinase 7 (PTK7), which is overexpressed on the cell membrane surface in BC cells, a novel targeting system protein tyrosine kinase 7 aptamer-Gemcitabine conjugate (PTK7-GEMs) was designed and synthesized using a specific PTK7 aptamer and GEM through auto-synthesis method to deliver GEM against BC. In addition, the antitumor effects and safety evaluation of PTK7-GEMs was assessed with a series of in vitro and in vivo assays.

**Results:**

PTK7-GEMs can specifically bind and enter to BC cells dependent on the expression levels of PTK7 and via the macropinocytosis pathway, which induced cytotoxicity after GEM cleavage from PTK7-GEMs respond to the intracellular phosphatase. Moreover, PTK7-GEMs showed stronger anti-tumor efficacy and excellent biosafety in three types of tumor xenograft mice models.

**Conclusion:**

These results demonstrated that PTK7-GEMs is a successful targeted aptamer-drug conjugates strategy (APDCs) to treat BC, which will provide new directions for the precision treatment of BC in the field of biomarker-oriented tumor targeted therapy.

**Supplementary Information:**

The online version contains supplementary material available at 10.1186/s40824-022-00328-9.

## Background

Among urological tumors, bladder cancer (BC) is one of the most common, which can be typically classified as muscle-invasive BC (MIBC) or non-muscle-invasive BC (NMIBC) [1]. The combination of surgery and chemotherapy is typically used to treat BC. Although advances in surgical treatments and combination chemotherapy (cisplatin and gemcitabine) protocols have improved median survival, approximately 50% of patients with BC develop recurrent or metastatic disease [2]. Many treatment strategies have been explored to manage BC, such as new combination chemotherapy, immunotherapy, and gene therapies [3]. Chemoresistance and side effects of classical anticancer drugs occur during treatment, immune checkpoint inhibitors (ICIs) induce their off-target effects [4], antibody-drug conjugates (ADCs) immunogenicity risk and the increased incidence of drug resistance remain challenges [5]. Therefore, a new targeting strategy to treat BC is urgently needed.

Recently, a biomarker-targeted functional nucleic acid aptamer-drug conjugates (APDCs) strategy was developed, in which aside chain of a small molecular drug (SMD) is conjugated to an aptamer through smart-responsive link that specifically recognizes cancer cells surface biomarker to deliver the cytotoxic anticancer drugs [6]. Nucleic acid aptamers are short single-stranded (ssDNA or ssRNA) molecules produced by systematic evolution of exponentially enriched ligands (SELEX) [7]. Single-stranded nucleic acid molecules are irregularly folded to form stable secondary and tertiary spatial structures specifically binding the target biomarker to distinguish normal cells from tumor cells, such as hairpins, false knots, convex rings, and G-tetramers [8–11]. Furthermore, aptamer is non-immunogenic and have the advantage of easy synthesis and modification, and allow site-specific coupling of fluorescent dyes, radionuclides, drugs, and pharmacokinetic regulators [12–20]. Therefore, APDCs not only dramatically improve specific transportation by biomarker targets, but also solve the problem of high solubility in water and weak affinity of anticancer agents [21]. For example, evidence shows that the conserved biomarker transmembrane protein tyrosine kinase 7 (PTK7), a pseudokinase family of receptor tyrosine kinases family member, is overexpressed in colon cancer, breast cancer, lung cancer, and esophageal cancer compared with that in normal tissues, and has vital functions in tumorigenesis and progression [22–26]. The catalytic inactivity of PTK7 allowed the PTK7specific DNA aptamer, Sgc8, which effectively mediates cell specific binding and internalization on the cell surface membrane of PTK7 overexpressing, to be screened by CELL-SELEX [27]. Many studies have shown that Sgc8 is a promising cell-specific intracellular drug delivery carrier and no cytotoxicity [28–30].

There have been no reports about the PTK7 expression level or any nucleic acid aptamer-drug conjugates based on biomarkers that target to BC. Gemcitabine (GEM), the main first-line chemotherapy for BC treatment, is a kind of antimetabolic cytotoxin that interferes with the incorporation of nucleotides into DNA [31, 32]. However, GEM and small molecular anticancer drugs have the characteristics of hard solubility and weak affinity to tumors, often produce limited clinical efficacy and serious adverse side effects.

**Scheme 1:**
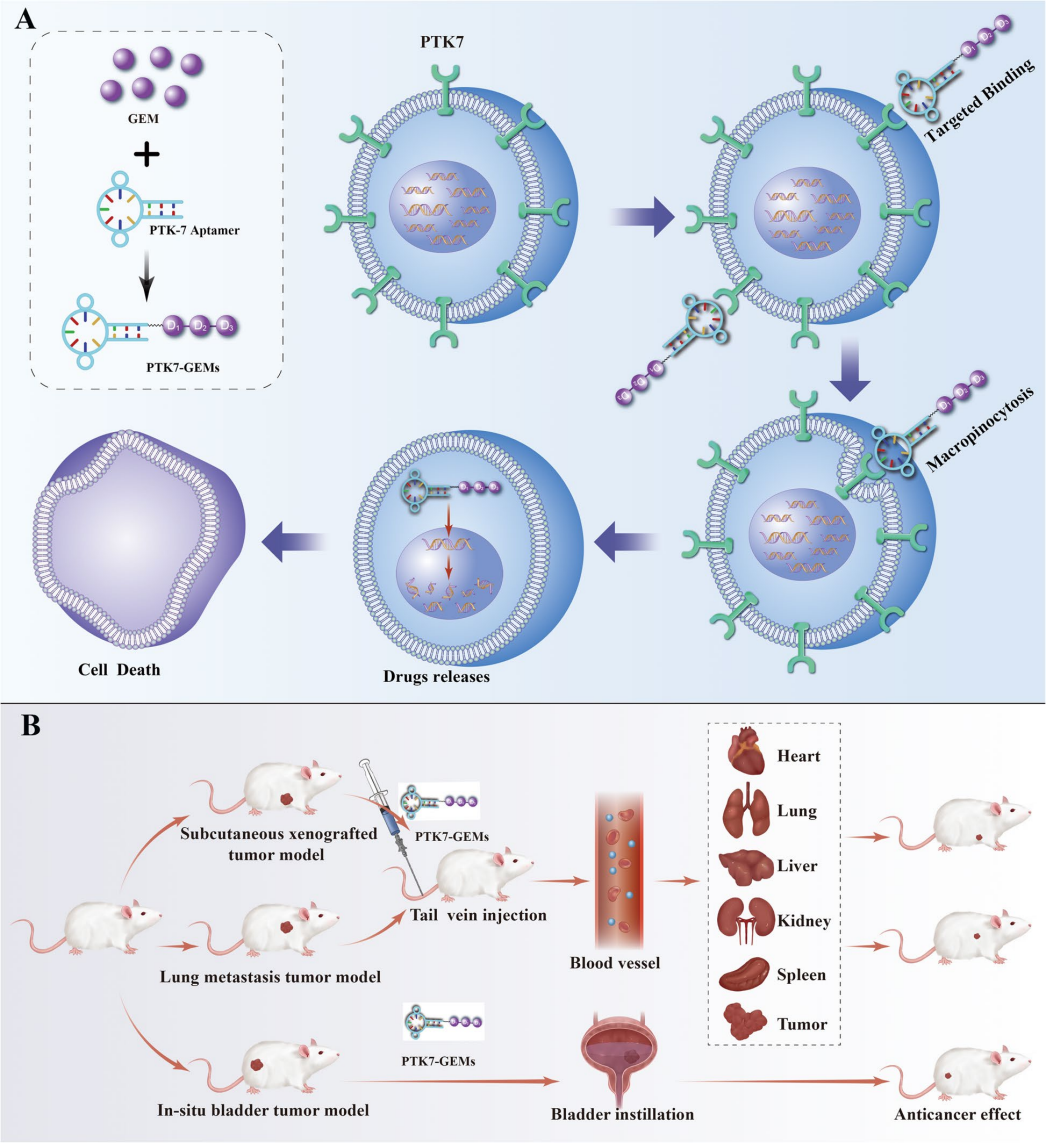
Schematic diagram and application of PTK7-GEMs. **A** Schematic diagram showing GEM is transformed into a modular drug-type nucleotide and then combined with PTK7-Aptamer to produce PTK7-GEMs. **B** Application and anti-tumor effect of PTK7-GEMs in three different tumor models

In this study, we first identified PTK7 is a biomarker in BC because of PTK7 overexpression in BC tissues and BC cells. Then, a novel targeting system, PTK7-GEMs, was synthesized via phosphodiester bond between Sgc8 and three GEM molecules. PTK7-GEMs could target BC and fully release the loaded GEM via the action of intracellular phosphatase. The released GEM could exert strongly cytotoxicity to induce BC cells death (Scheme 1A). Moreover, the anti-tumor efficacy and biosafety of PTK7-GEMs was verified in vivo using three human tumor xenograft models (subcutaneous tumor, lung metastasis tumor, and bladder *in situ* tumor) (Scheme 1B).

## Methods and materials

### General reagents

The washing buffer (5$\times$10^− 3^ м MgCl_2_ and 4.5 g L^− 1^ glucose in Dulbecco’s PBS) was obtained from Sigma-Aldrich (St. Louis, MO, USA), and the binding buffer was composed with yeast tRNA (0.1 mg mL^− 1^, Sigma-Aldrich) and bovine serum albumin (BSA; 1 mg mL^− 1^ (ThermoFisher Scientific, Waltham, MA, USA) in washing buffer, which was used to reduce background binding. Super GelRed was purchased from US Everbright® Inc (Sayreville, NJ, USA). Beijing Labor Technology Co, Ltd. provided the Cell Counting Kit-8 (CCK-8) (Beijing, China; cat. no. 21,162,196). Protein markers were purchased from Sangon Biotech Co. Ltd. (Shanghai, China). The anti-PTK7 rabbit monoclonal antibody was purchased from Abcam (Cambridge, MA, USA). The Alexa Fluor 488-labeled goat anti-mouse secondary antibody, Hoechst 33,342, lysotracker, radioimmunoprecipitation assay (RIPA) lysis buffer, phosphatase inhibitors cocktail, protease inhibitors, and annexin V-FITC Apoptosis Detection Kit (no. 011821210207) were all purchased from Beyotime Biotech Inc (Jiangsu, China). All chemicals for synthesis and purification were purchased from Energy Chemical (Shanghai, China). All tubes and plates for cell culture were purchased from NEST Biotechnology (Jiangsu, China). Unless otherwise stated, all other bioreagents were purchased from Sigma.

### Cell culture

The human BC cells (BIU87, 5637, T24, EJ, RT4, J82, UM-UC-3, TCCSUP), and SVHUC-1, a normal bladder uroepithelial cell line, were purchased from the ATCC (Rockville, MD, USA). 5637 and SV-HUC-1 cells were cultured in RPMI-1640 and F12K medium, respectively. Other cells were cultured in Dulbecco’s Modified Eagle’s Medium (DMEM), both including 10% FBS. Dulbecco’s Phosphate-Buffered Saline (DPBS) without Ca^2+^ and Mg^2+^ was used to wash the cells.

### Patient information and tissue specimens

The tumor specimens were obtained from paraffin blocks of 148 primary BC tissues, which were diagnosed histologically as bladder urothelial carcinoma at the Third Xiangya Hospital of the Central South University and Sun Yat-sen University Cancer Center (2002–2012), eighty-five cases of normal bladder mucosa in paraffin blocks from adjacent non-neoplastic bladder tissue of the same patients with BC as parallel control. Previously published criteria were used to select the cases [33]. Moreover, 20 pairs of fresh BC tissues and their matched normal bladder mucosa tissues were frozen and stored in liquid nitrogen until further experimentation.

### Immunohistochemistry (IHC)

While paraffin-embedded sections on slides were cleared using xylene and then rehydrated using ethanol-water solutions, the sections were heated for 3 min at 120 °C in 0.01 м citrate buffer (pH 6.0) to retrieve the antigen. The slides were immersed in 3% hydrogen peroxide for 20 min to block endogenous peroxidases and then the tissues were blocked for 20 min using universal blocking serum. The tissue sections were then incubated with anti-PTK7 antibodies (Abcam; 1:300 dilution) for 2 h at room temperature and then incubated with biotin-labelled secondary antibody and streptavidin-peroxidase for 20 min each. Finally, signal development was achieved using 3,3-diaminobenzidine (DAB). Substituting a normal murine IgG for the primary antibody comprised the control. Positive controls comprised known immunostaining positive slides. The total immunostaining scores for PTK7 were determined as the sum of percent of positively stained tumor cells and the staining intensity. The percent of positively stained tumor cells was scored as 0 (< 5% positive), 1(5–25%), 2 (25–50%), 3 (50–75%), and 4 (> 75%). The staining intensity was scored as 0 (absence staining), 1 (weak staining), 2 (moderate staining), and 3 (strong staining). Double-blind conditions were when determining the percent of positively stained tumor cells and the staining intensity. The PTK7 immunostaining score was calculated as the value of the percent of positively stained tumor cells plus the staining intensity score, and ranged from 0 to 12. PTK7 levels were defined as: ‘−’ (score 0–3), ‘+’ (score 4–6), ‘++’ (score 7–9), and ‘+++’ (score ≥ 10). To groups of patients with BC were then classified: low PTK7 expression (− and +) and high PTK7 expression ( + + and +++).

### Immunofluorescence (IF) staining

Cells (5 ⋅ 10^4^ cells mL^− 1^, with or without treatment) were seeded onto glass coverslips in six-well plates for IF staining. Following treatment for the specified times, 2% paraformaldehyde was used to fix the cells for 5 min, which were then washed with Tween-20 (PBST). Thereafter, the slides were incubated with antibodies against PTK7 (Abcam; 1:100 dilution) for 1 h at room temperature, followed by incubation for 1 h with secondary antibodies, before being subjected to 4′,6-diamidino-2-phenylindole (DAPI) counterstaining for nuclear observation.

### Quantitative real-time reverse transcription polymerase chain reaction (qRT-PCR)

Total RNA was extracted from clinical samples using the TRIzol reagent (Invitrogen, Waltham, MA, USA) and was reversed transcribe to cDNA following the vendor’s protocols (RT-for-PCR kit, Clontech Laboratories, Mountain View, CA, USA). The resultant cDNA corresponding to the *PTK7* mRNA was measured using quantitative real-time PCR (qPCR) using a SYBR Green PCR Kit (Applied Biosystems, Foster City, CA, USA) and a LightCycler480 384-well PCR system (Roche Diagnostics, Basel, Switzerland). The *ACTB* gene (encoding β-actin) comprised the internal control for PTK7. *PTK7* primers were 5′ACACTTCGTTGCCACATTGAT-3′ (forward) and 5′CAGCAGGAATACAGCCCAC-3′ (reverse). *ACTB* primers were 5′CATTAAGGAGAAGCTGTGCT-3′ (forward) and 5′GTTGAAGGTAGTTTCGTGGA-3′ (reverse). The relative expression of the gene in each sample was averaged and compared using the cycle threshold (Ct) method. The 2–Ct method was used to calculate the fold changes in mRNA levels.

### Western blotting

Total proteins were extracted using RIPA lysis buffer, separated by 15% SDS-PAGE in the mini trans-blot apparatus (Bio-Rad Laboratories, Hercules, CA, USA) and transferred onto polyvinylidene difluoride membranes (Roche, Basel, Switzerland). The membranes were blocked for 1 h at room temperature in TBS-0.1% Tween 20 with 5% nonfat dry milk. Thereafter, the membranes were reacted with rabbit polyclonal antibody recognizing PTK7 (Abcam; 1:500) or ACTB (quality control, Santa Cruz Biotechnology, Santa Cruz, CA, USA; 1:800) overnight at 4 °C. After washing thrice with TBS-0. % Tween 20, the membranes were incubated with horseradish peroxidase (HRP)conjugated goat anti-rabbit IgG antibody (Cell Signaling Technology, Danvers, MA, USA; 1:3000 dilution) at room temperature for 1 h. Immunoreactive protein bands were visualized using a Luminata Crescendo Western HRP substrate (Millipore, Billerica, MA, USA).

### Oligonucleotide synthesis and purification

According to the reported method and synthesis procedure shown in Figure S1 [12], Li_2_CO_3_ (900 mg) and lutidine (1350 mg) were first added to compound 1 (gemcitabine, 269 mg, ~ 1.0 mmol, purity 97%) that was suspended with anhydrous CH_2_Cl_2_ (100 mL). 4,4’-dimethoxytrityl tetrafluoroborate salt (DMTrBF4) (~ 460 mg, 1.12 mmol) was added in portion, 150 mL of CH_2_Cl_2_ was added to the reaction, and the diluted solution was washed with saturated NaCl solution and dried over anhydrous Na_2_SO_4_. After removal of the solvent, the residue was purified using flash column giving compound 2 (~ 400 mg, yield 73%; M + H^+^ = 543.3). Compound 2 (264 mg, ~ 0.5 mmol) was resuspended in 40 mL CH_2_Cl_2_, added with N, N-Diisopropylethylamine (DIEA) (650 mg, 5.0 mmol) and cooled at 0 °C. N N-diisopropylchlorophosphoramidite (596 mg, 2.44 mmol) was added and the reaction was monitored by thin-lay chromatography (TLC). When the starting material had disappeared, the reaction solution was diluted using CH_2_Cl_2_ (~ 100 mL) and rinsed using saturated NaHCO_3_ and saturated NaCl. Then, the obtained products were dried with anhydrous Na_2_SO_4_, the dried solution was concentrated, and the residue was purified using a flash column, giving GEM phosphoramidite 3 (white powder, ~ 380 mg, 80% yield; MW calculated for 950.03; M + Na^+^ = 973.09), the MS, ^1^ H-NMR, and ^13^ C-NMR were correct based the calculated structure (Fig.S2-S4). Following the PTK7-GEMs sequence, synthesis was run automatically according to the requirements of the DNA synthesizer (PolyGen GmbH, Langen, Germany). After auto-synthesis, the crude products were treated with reference to the reported standard [34]. Briefly, the DNA products were treated with about 400 µL of 28% ammonium hydroxide to cleave the CpG of the oligonucleotides at 65 °C for 30 min. The cleaved DNA was mixed with 1 mL of ice-cold ethanol and 40 µL of 3 mol/L NaCl, and the oligonucleotides were precipitated at for 1 h at -20 °C, followed by centrifugation for 20 min at 4 °C and 12, 000 rpm; the pellet was retained. The precipitate was dissolved using 400 µL of 0.1 mol/L triethylamine acetate (TEAA) for subsequent HPLC purification using a C18 column. The DNA product was lyophilized, resuspended in sterilized ultrapure water, and then desalted using a desalt mini column. The FITC- and Cy5-labeled strands were synthesized and purified. All DNA sequences were quantified and stored in sterilized water for further experiments.

### Polyacrylamide gel electrophoresis

PTK7-GEMs (5$\times$10^− 6^ м) was incubated in 10% FBS-supplemented RPMI-1640 medium or nude mice serum at 37 °C for 0, 1, 2, 4, 8, 12, 16, and 24 h respectively. The samples were then denatured in for 5 min at 95 °C f and 10 µL aliquots were subjected to 12% polyacrylamide gel electrophoresis at 110 V for 45 min. Super GelRed (US Everbright® Inc) was used to stain the gel for imaging.

### Binding assay

A total of 3 ⋅ 10^5^ cells (5637 and SV-HUC-1 cells) were washed using washing buffer via centrifugation at 1000 rpm for 3 min and then incubated with 200 $\times$10^− 9^ м FITC-labeled DNA aptamers (PTK7-GEMs, LIB-GEMs, PTK7 and LIB) at 4 °C in 200 µL of binding buffer for 1 h for a binding assay. For the competitive binding assay, label-free aptamer (PTK7, LIB) was preincubated with 5637 cells for 1 h, and then 200 $\times$10^− 9^ м FITC labeled aptamer (PTK7-GEMs and LIB-GEMs) were added and incubated at 4 °C for another 1 h respectively. The samples were washed thrice using washing buffer, collected, and resuspended in 500 µL of binding buffer for BD FACSVerse™ flow cytometry (BD Biosciences, San Jose, CA, USA).

### Cytotoxicity assay

Bladder cancer cell lines or SV-HUC-1 cell lines were respectively seeded in a 96-well plate (3000 cells/well) and incubated for 24 h for adherence. Thereafter, 100 µL of RPMI-1640 or DMEM complete cell medium containing different samples (PTK7-GEMs, LIB-GEMs, GEM) at different concentration (25, 50, 100, 200, 400, and 800 $\times$10^− 9^ м) were added and incubated for 8 h, untreated cells as the control, The cell medium was then removed, and the cells were washed with DPBS. After that, 100 µL of fresh complete cell medium were added to the cultured cells for another 72 h. For CCK-8 analysis, the cell medium was substituted with 100 µL of fresh complete cell medium with 10% CCK-8. After incubating for the appropriate time, cell viability was determined by measuring absorbance at 450 nm in a Synergy 2 Multi-Mode Microplate Reader (Bio-Tek, Winooski, VT, USA).

### Flow cytometry determination of cell apoptosis

Five thousand, six hundred thirty-seven cells were added in 6-well culture plates, followed by overnight incubation for adherence. Adherent cells were treated using 50 $\times$10^− 9^ м PTK7-GEMs, LIB-GEMs, GEM for 8 h and then incubated for 72 h with Complete medium, respectively, with untreated cells comprising the control. Thereafter, cells were washed with PBS, harvested, and resuspended in 5 µL of Annexin-V FITC with 195 µL binding buffer and 5 µL of 7-Aminoactinomycin D (7-AAD), according to the supplier’s guidelines (Annexin V-FITC Apoptosis Detection Kit, Beyotime Co.), and then incubated for 15 min in the dark at room temperature to analysis in flow cytometry (Cytek DxP Athena; Cytek, Fremont, CA, USA).

### Drug release kinetic profiling

For GEM release in cell lysates, 5637 cells (5 ⋅ 10^6^) were lysed using RIPA lysis buffer (0.1% SDS, 1% sodium deoxycholate, 1% Triton X-100, 150 $\times$10^− 3^ м NaCl, and 50 $\times$10^− 3^ м Tris, obtained from Beyotime Biotechnology Co.) without inhibitors (or with 50 ⋅ phosphatase inhibitors cocktail), and the clarified lysate obtained was treated with 50 $\times$10^− 6^ м PTK7-GEMs for 0, 0.5, 1, 2, and 4 h at 37.0 °C, respectively. Thereafter, the lysates were added with acetonitrile, vortexed for 3 min, and centrifugation for 10 min at 12, 000 rpm (using the same treatment condition as that described below). For GEM release in response to other responsive factors, all experiments underwent extraction to rule out interference. Extracted supernatants were analyzed using HPLC to quantify the release of GEM from PTK7-GEMs. The characteristic absorbance of DNA at 260 nm was determined.

### Endocytic pathway study

Bladder cancer cell lines or SV-HUC-1 cell line were added to a 15 mm confocal dish (NEST Biotechnology) and grown overnight for adherence before the experiments. For the binding colocalization assay, 5% BSA was used to block cells for 1 h. After aspirating off the BSA. Cells were incubated with 200 $\times$10^− 9^ м of cy5-labeled PTK7-GEMs and Alexa Fluor 488-labeled endocytic markers (25 µg mL^− 1^ dextran, 25 µg mL^− 1^ transferrin and 5 µg mL^− 1^ transferrin), DAPI then was added during the final 15 min of the incubation, after 2 h of incubation, the cells were washed with wash buffer and visualized by confocal microscopy. next, cells were treated with corresponding pharmacological inhibitor: 1 $\times$10^− 3^ м methyl-β-cyclodextrin (the caveolae pathway), 0.1 $\times$10^− 3^ м chlorpromazine (the clathrin pathway), and 0.1 $\times$10^− 3^ м EIPA (the macropinocytosis pathway), after preincubation for 2 h, 200$\times$10^−9^ м cy5-labeled PTK7-GEMs and Alexa Fluor 488-labeled endocytic markers was added and then incubated for 2 h in the presence of the inhibitors, Cells were washed with wash buffer, then visualized by confocal microscopy.

### Lysosomal escape

Confocal microscopy was used to study the Cy5 labeled PTK7-GEMs endosomal escape. 5 × 10^3^ bladder cancer cells seeded and incubated overnight into CLSM dishes at 37 °C, which were then incubated with 200 $\times$10^− 9^ м PTK7-GEMs-Cy5 for 2 h. The cells were then washed with PBS three times and incubated with 250 $\times$10^− 9^ м Lyso Tracker Green for 30 min and with 100 ng/ml DAPI for 15 min. These cells were then observed immediately using CLSM at 2 and 4 h respectively.

### Calcein-AM/PI double staining assay

Bladder cancer cells (5 × 10^3^ cells/well) were seeded into a 96-well plate overnight at 37 °C, and incubated with PBS, PTK7-GEMs respectively for 4 and 8 h. Subsequently, the medium in each well was discarded, then Calcein-AM (2 µmol/mL) and PI (50 µg/ mL) was added to stain in the dark for 20 min, followed by observation of staining under fluorescence microscope.

### Biological imaging of PTK7-GEMs in vivo

About 8 × 10^6^ cells in ~ 100 µL DPBS were subcutaneously injected in right underarm of each female mouse (BALB/c nude mice 4–5 weeks). When the tumor volume approximately 700 mm^3^, cy5-labeled PTK7-GEMs or LIBGEMs (50 $\times$10^− 6^ м, 200 µL) were injected into the tail veins of mice bearing tumors. The IVIS Lumina XR optical imaging system (Perkin-Elmer, Waltham, MA, USA) was used to perform imaging at various times before or after injection. After 4 h of imaging, the mice were euthanized and dissected. Tumor tissue imaging, including the main visceral organs (heart, liver, spleen, lung, and kidney) was carried out to assess ex vivo bio-distribution.

### Anti-tumor activity of PTK7-GEMs in xenotransplanted subcutaneous tumors

About 8 × 10^6^ of 5637 cells in ~ 100 µL DPBS were subcutaneously injected in right underarm of each female mouse (BALB/c nude mice 4–5 weeks). When the tumor size reached 100–200 mm^3^, PBS, PTK7, LIB, GEM, LIB-GEMs, and PTK7-GEMs (equivalent GEM concentration = 16 mg kg^− 1^) were administered to the mice in the six groups (*n* = 6 mice per group) by i.v. every other 2 days for eight times in total. All materials were dissolved in PBS. After the last dose, the mice were subjected to humane killing. Blood samples were taken from the mice for biochemical and hematological analyses. We excised the tumors and subjected them to immunohistochemical, immunofluorescent, and histological examinations.

### Anti-tumor activity of PTK7-GEMs in a mouse model of lung metastasis induced via tail vein injection

To investigate PTK7-GEMs in vivo antitumor activity, a SCID mouse model of lung metastasis was created by tail vein injection of bladder cancer cells. The tail veins of BALB/c nude mice bearing tumors were injected with 8 ⋅ 10^6^ bladder cancer cells in 100 µL of precooled PBS. Four weeks later, the mice were placed randomly into six groups: PTK7, LIB, GEM, LIB-GEMs, or PTK7-GEMs (equivalent GEM concentration = 16 mg kg^− 1^), which were administered to the mice in the six groups via i.v. once a week for 5 weeks. The control group was administered with PBS. After 5 weeks of treatment, the mice were sacrifice humanely using CO_2_ and their lung tumor tissues were excised for subsequent histological analysis.

### Anti-tumor activity of PTK7-GEMs in an in-situ bladder cancer model

To further study the in vivo antitumor activity of PTK7-GEMs, we created a rat model of *in-situ* bladder cancer. Ether inhalation was used to anesthetize female SD rats, followed by infusion of their bladders with 0.2 ml of 10 mg mL^− 1^ N-methyl-Nitrosurea (MNU; Sigma) using a 22-gauge angiocatheter once every two weeks, five times. Postcatheterization, spontaneous micturition was avoided by keeping the rats anesthetized for approximately 45 min. After the successful induction of tumors (about 16 w), rats (*n* = 60) were placed in six groups, each comprising ten rats. After anesthesia, the rats’ bladders were instilled with PBS, PTK7, LIB, GEM, LIB-GEMs, or PTK7-GEMs (with the equivalent GEM concentration of 5 mg kg^− 1^). Spontaneous micturition was avoided by keeping the rats anesthetized for approximately 45 min. Treatments were given once each week for 5 w. The rats were subjected to human sacrifice at 2 days after the last dose. We dissected out the rats’ bladders, which were weighed, 4% paraformaldehyde-fixed for 24 h, paraffin-embedded, and subjected to histopathological examination. At the bladder midpoint, we cut transverse sections, followed by staining with H&E.

### Data analysis

GraphPad Prism 8.0 (GraphPad Inc., La Jolla, CA, USA) was used to display differences between two groups (Student’s t test) or among three or more groups. For colocalization analysis of confocal images, ImageJ software (NIH, Bethesda, MD, USA) was used to calculate Pearson’s correlation coefficient (PCC) and Manders’ coefficient (M1). The association between PTK7 expression and clinicopathological variables was assessed using a chi-squared test. Survival curves were plotted with the aid of the Kaplan–Meier method. Univariate and multivariate examinations were carried out using the Cox proportional hazards regression model. Statistical analyses were carried out using a combination of Tuckey’s test and one-way ANOVA, the mean ± SEM or SD were utilized to present the value, the significance was indicated with *P*-value < 0.05.

## Results

### The establishment of biomarker PTK7 in BC

**Fig. 1:**
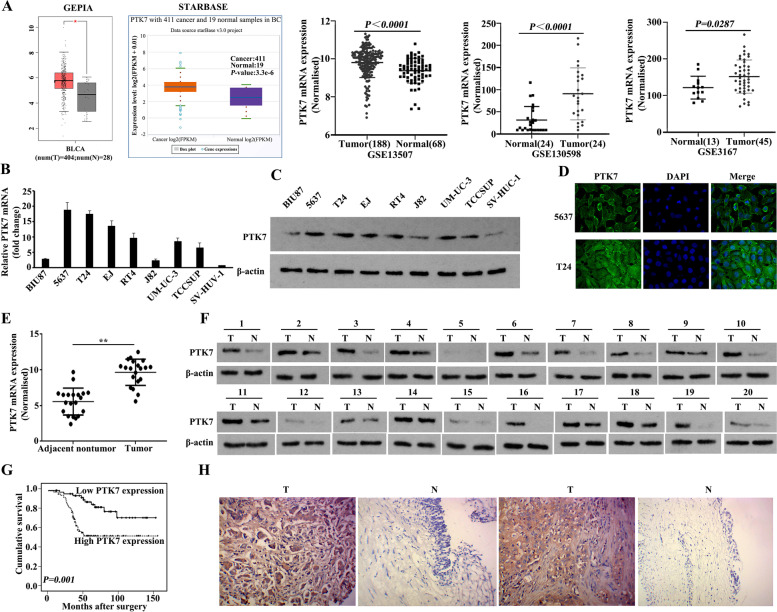
The establishment of biomarker PTK7 in bladder cancer. **A** The high expression of PTK7 in BC was predicted using GEPIA, STARBASE, and GSE databases. **B**,** C** The relative expression of PTK7 mRNA and protein in BC cell lines and the normal bladder uroepithelial cell line SV-HUC-1, as assessed using qRT-PCR and western blotting. **D** PTK7 immunofluorescence was observed primarily in the BC cell membrane. **E**,** F** PTK7 expression was higher in BC tissues than in adjacent normal bladder urothelial tissues according to qRT-PCR and western blotting analyses. **G** Univariate analyses shown Kaplan–Meier curves for patients expressing low levels of PTK7 versus those expressing high levels of PTK7 in BC tumors. **H** Representative images of immunohistochemistry showing high expression of PTK7 in a BC tissue sample and low of PTK7 expression in a sample of adjacent normal bladder urothelial tissue. Original magnification, ⋅ 200

To predict and confirm that PTK7 could be a BC-enriched biomarker, we carried out an objective analysis by querying the GEPIA, STARBASE and GSE databases, which showed high PTK7 overexpression in BC (Fig. 1A). Thereafter, we determined the PTK7 mRNA and protein levels in various BC cell lines (BIU87, 5637, T24, EJ, RT4, J82, UM-UC-3, and TCCSUP) and in a normal bladder uroepithelial cell line (SV-HUC-1). These results showed that PTK7 was overexpressed to different levels in BC cells, but showed negative expression in SV-HUC-1 cells (Fig. 1B and C). Furthermore, immunofluorescence analysis exhibited strong PTK7 staining on the BC cell membrane (Fig. 1D). Moreover, from the analysis of 20 pairs of clinical BC tissues, we observed marked upregulation of PTK7 transcription and translation in BC tissues compared with that in adjacent normal urothelial tissues, which is accordance with the analysis of quantitative real-time reverse transcription PCR (qRT-PCR) and western blotting respectively (Fig. 1E and F). High PTK7 expression was observed in 90/148 (60.81%) of BC tissues and in 12/85 (14.12%) of normal urothelial bladder epithelial tissues (*P* < 0.001, Fig. 1G and H). The expression of PTK7 was statistically analyzed for its correlation with the clinicopathological features of BC (Additional file 1: Table S1). High expression of PTK7 correlated strongly with advanced T stage (*P* = 0.018) and advanced N stage (*P* = 0.009) in the cohort of 148 cases of BC. Kaplan–Meier analysis indicated that poor patient survival correlated positively with high PTK7 expression in BC (*P* = 0.001, Fig. 1H; Additional file 1: Table S2). Finally, multivariate survival analysis showed that the PTK7 expression level was an independent prognostic factor for poor survival (hazard ratio (HR): 3.105, 95% confidence interval (CI): 1.561–6.177, *P* = 0.001, Additional file 1: Table S3). Together, these results demonstrated that PTK7 is a biomarker for BC.

### Synthesis of aptamer PTK7-GEMs Conjugate

To construct the aptamer PTK7-GEMs conjugate, the GEM phosphoramidite building block was prepared by one-step synthesis and coupling of commercial GEM and N, N-diisopropylchlorophosphamide, with a yield of 80.0% (Additional file 1: Fig. S1-S4). Three GEM phosphoramidites were incorporated into the PTK7 aptamer Sgc8 sequence to obtain the PTK7-GEMs conjugate using automated solid-phase DNA synthesis. Briefly, the sequences were synthesized from the 3’ to the 5’ end of the oligonucleotides, the CpG of the first base of the target sequence 3’ was selected as the solid phase carrier, DMT protection of first base 5’-OH was removed with trichloroacetic acid, and after activation, trichloroacetic acid was eluted using anhydrous acetonitrile, followed by tetrazole and phosphoramidite addition. The phosphoramidite activated by tetrazole reacts with the 5’ OH to form a phosphite bond, and then trivalent phosphorus was oxidized to pentavalent phosphorus by I_2_ to form a stable phosphodiester bond. Acetyl blocks the small amount of 5’-OH that does not participate in the coupling reaction, and each cycle reaction adds one base until the target sequence is completed (Additional file 1: Fig. S5-S9**)**. Further, PTK7-GEMs conjugates and the other conjugates were purified by high performance liquid chromatography (HPLC) (Additional file 1: Table S4; Fig. S10-S13).

### The stability of PTK7-GEMs in serum and targeting in vitro

**Fig. 2:**
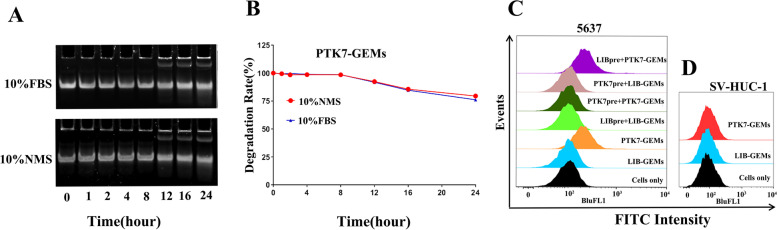
Serum stability and cell binding ability of PTK7-GEMs. Serum stability analysis of PTK7-GEMs using 12% polyacrylamide gel electrophoresis (PAGE). **A** PTK7-GEMs were incubated for 0, 1, 2, 4, 8, 12, 16, and 24 h 10% FBS-supplemented or 10% NMS-supplemented RPMI-1640 medium, respectively. **B** The abscissa indicates that the same sample of PTK7-GEMs was incubated in 10% NMS (red) and 10% FBS (blue) for 0, 1, 2, 4, 8, 12, 16, and 24 h respectively, and the ordinate indicates the respective degradation percentage of the samples. The binding ability of PTK7-GEMs to PTK7 overexpressing cells at 4 °C, as determined using flow cytometry. **C**,** D** The binding ability of various FITC-labeled samples to 5637 cells and SV-HUC-1 cells

To assess the stability of PTK7-GEMs in biological medium, Roswell Park Memorial Institute (RPMI)-1640 cell culture medium supplemented with 10% fetal bovine serum (FBS) and 10% nude mice serum (NMS) was prepared to simulate the growth environment of tumor respectively [35]. The results showed that PTK7-GEMs did not degrade in the first 8 h in 10% FBS and 10% NMS (Fig. 2A), and the degradation was about 20% in 24 h (Fig. 2B). Therefore, the synthesized PTK7-GEMs could maintain good stability, which ensured the ideal blood concentration of PTK7-GEMs, and increased the chance of interaction between tumor tissue and aptamer-targeted drugs. Then, while PTK7-GEMs (fluorescein isothiocyanate, FITC) and its control sequence, LIB-GEMs (FITC), was incubated with the PTK7-high expression 5637 cell line and the PTK7 negative expression SV-HUC-1 cell line respectively, we can observe that PTK7-GEMs (orange) showed a higher fluorescence intensity than LIBGEMs (blue) on 5637 cells (Fig. 2C), indicated that PTK7-GEMs can specifically bind to 5637 cells than LIB-GEMs, which verify the targeting of PTK7-GEMs through cell membrane surface PTK7 to BC cells. And the competition experiment further verified the above results (Fig. 2C). These results suggest that PTK7-GEMs can target the 5637 cells line with high binding affinity to the PTK7 protein. To the normal bladder uroepithelial cell line SV-HUC-1 (Fig. 2D), PTK7-GEMs and LIB-GEMs showed similar results to the cells only control, with no obvious fluorescence intensity, demonstrating that PTK7-GEMs could not target SV-HUC-1 cells because of its lack of PTK7 protein expression. These results confirmed that PTK7-GEMs can distinguish target cell lines through PTK7 overexpression.

### PTK7-GEMs selectively inhibits BC cell proliferation

**Fig. 3:**
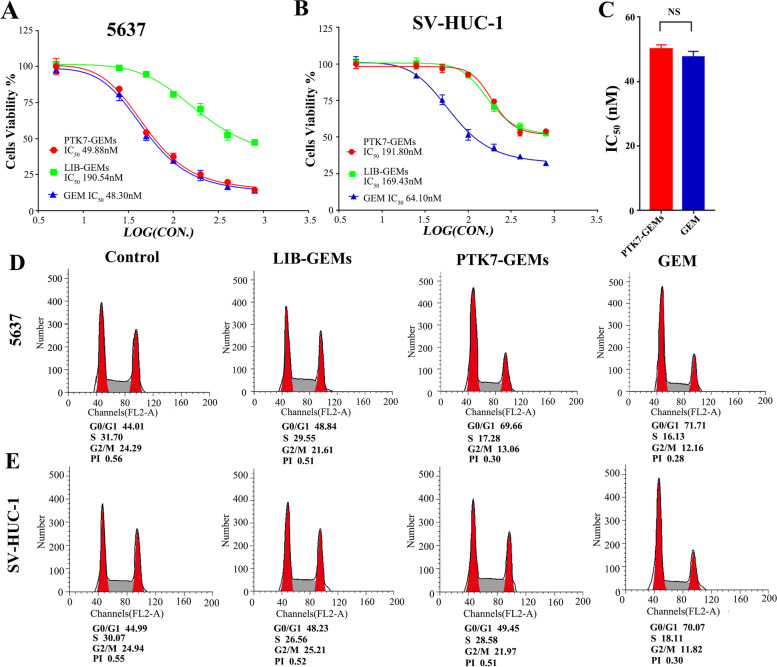
CCK-8 assays of PTK7-GEMs cytotoxicity. **A**, **B** Viability of 5637 cells and SV-HUC-1 cells respectively treated with LIB-GEMs, GEM or PTK7-GEMs evaluated by CCK8 assay. **C** IC_50_ of PTK7-GEMs and GEM against 5637 cells (NS, not significant). Data represents the mean ± SEM, *n* = 3. **D**,** E** Cell cycle distributions of 5637 and SV-HUC-1 cells treated with LIB-GEMs, GEM or PTK7-GEMs respectively for 8 h and then incubated for 72 h with complete medium before being tested using flow cytometry

The CCK-8 assay was used to evaluate the cytotoxicity of PTK7-GEMs toward bladder cancer cell line (5637) and a normal bladder uroepithelial cell line (SV-HUC-1). The data showed that GEM does not have a good selectivity for 5637 and SV-HUC-1 cells, because the IC_50_ values of these two cells were 48.3$\times$10^−9^ м and 64.1 $\times$10^− 9^ м (Fig. 3A and B) respectively. By contrast, PTK7-GEMs showed four times cytotoxicity difference between the 5637 cells and the SV-HUC-1 cells line (IC_50_ 49.88$\times$10^−9^ м vs. 191.8$\times$10^−9^ м, Fig. 3A-C). In addition, the maximum inhibition rate of PTK7-GEMs and GEM was about 85% on 5637 tumor cell line, while that of PTK7-GEMs toward normal bladder uroepithelial cells was lower than 45%; similar result was observed for the cell cycle and the apoptosis rate (Fig. 3D and E; Additional file 1: Fig. S14A and B). Meanwhile, PTK7-GEMs showed strongly cytotoxicity against cell line T24, and the cytotoxicity was comparable to that of GEM (Additional file 1: Fig. S15A and B), which was attributed to the high expression of PTK7. These results verified the specific binding and targeting ability of PTK7-GEMs to bladder cancer cells and its low cytotoxicity toward normal cells, compared with LIB-GEMs and GEM. Moreover, PTK7-GEMs, a single aptamer carrying three drug molecules, showed a higher delivery efficiency, also PTK7-GEMs could accurately deliver anticancer drugs and exert its strongly cytotoxicity toward target tumor cells.

### Drug release mechanism in vitro

**Fig. 4:**
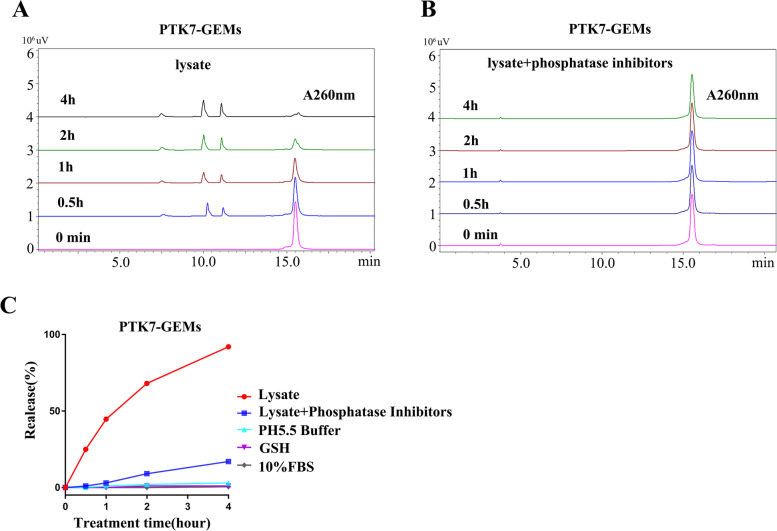
Investigation of the GEM release mechanisms from PTK7-GEMs. The profile of PTK7-GEMs release from **A** Crude cell lysate (untreated by inhibitors) or **B** Lysate of cells treated with phosphatase inhibitors, The DNA maximum absorption peak was measured at 260 nm. **C** Cumulative release of GEM when PTK7-GEMs were incubated with crude cell lysate, phosphatase inhibitor-treated cell lysate, buffer at pH 5.5, 10% FBS and 10 ⋅ 10^− 3^ м GSH respectively

To clarify the intracellular release of GEM from PTK7-GEMs, HPLC determination was carried out under different conditions, such as low-pH 5.5 buffer (mimicking the tumor microenvironment), 10% fetal bovine serum (cell culture medium), 10 × 10^− 3^ м glutathione (mimicking the tumor reduction microenvironment), and cell lysates or lysates with phosphatase inhibitors. The phosphodiester bond used as the bridge of the APDC, means that the drug was released via the action of an intracellular phosphatase [26, 29]. As shown in (Fig. 4A**)**, when a crude cell lysate from the 5637 cells line was used to treat PTK7-GEMs (retention time: 15.7 min) (without any inhibitor) at 37 ℃, timedependent release curves of PTK7-GEMs (retention time: 15.7 min) and aptamer fragments of different sizes (retention time: 7.5 min, 10.2 min, 11.3 min) were observed. In addition, the presence of phosphatase inhibitors significantly hindered the release of GEM from PTK7-GEMs within 4 h (Fig. 4B). Notably, in the presence of phosphatase, about 50% of PTK7-GEMs was degraded within 1 h and reached 75% within 2 h; however, in the presence of the phosphatase inhibitor, only about 20% of PTK7-GEMs was degraded within 4 h, while the other factors cause little or no obvious degradation within 4 h (Fig. 4C). These results supported the view that GEM release could be achieved via phosphatase-mediated degradation.

### Critical cellular events and molecular interactions

**Fig. 5:**
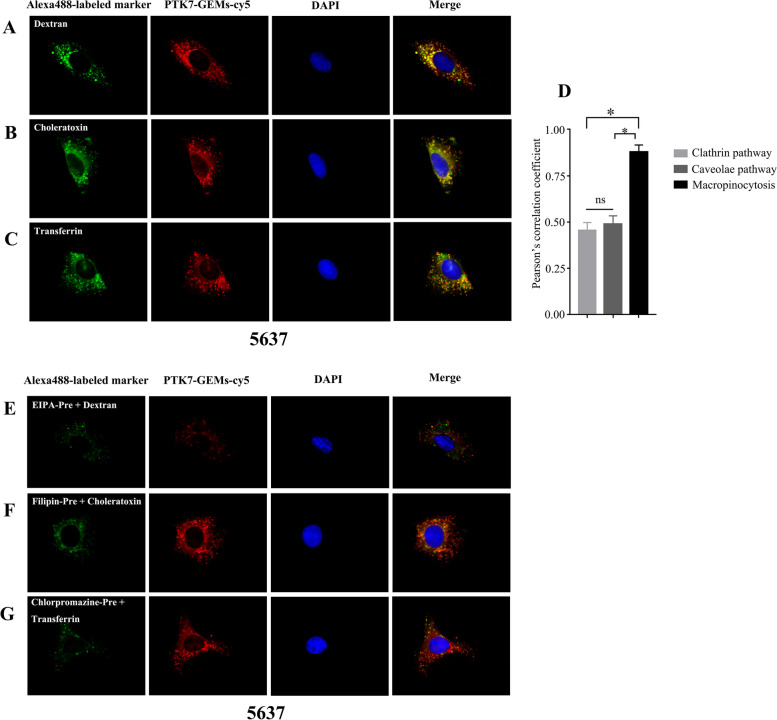
PTK7-GEMs internalization and trafficking in cells. Images generated using confocal microscopy revealing the co-localization of PTK7-GEMs-cy5 (red) with markers of endocytosis dextran (**A**), choleratoxin (**B**), transferrin (**C**) labeled with Alexa Fluor 488 respectively. **D** Pearson’s correlation coefficient analysis of PTK7-GEMs-cy5 and endocytosis marker co-localization in 5637 cells using Image J. Confocal microscopy images revealing the co-localization of PTK7-GEMs-cy5 (red) with EIPA (**E**) (inhibitor of macropinocytosis), Filipin (**F**) (inhibitor of the caveolae pathway), and Chlorpromazine (**G**) (inhibitor of the clathrin pathway) (green). DAPI was used to counterstain the nuclei (blue). Scale bar, 10 μm. The means ± standard deviation (*n* = 3 per group) is indicated using error bars. Each replicate was from a single biological experiment and 10 independent fields of view were chosen from quantification. *P < 0.05; NS = not significant

To observe whether the PTK7-GEMs was taken up by BC cells line through the endocytosis pathway, the 5637 cells were investigated with endocytosis markers labeled with Alexa Fluor-488 and subjected to confocal microscopy. Dextran (green) (a macropinocytosis marker, Fig. 5A) was significantly co-localized with PTK7-GEMs-cy5 (red) in 5637 cells, which different from choleratoxin (a caveolae mediated endocytosis marker, Fig. 5B) and transferrin (a clathrin mediated endocytosis marker, Fig. 5C) with PTK7-GEMs-cy5 (red), the pearson correlation coefficient of macropinocytosis was about 0.8 (Fig. 5D), which indicated that PTK7-GEMs-cy5 was mainly absorbed by 5637 cells via macropinocytosis. The co-localization between PTK7-GEMs-cy5 and dextran (Fig. 5E) was significantly reduced by the inhibitor (Fig. 5E, EIPA, a macropinocytosis inhibitor) compared with the other endocytosis inhibitor pretreatment (Fig. 5F, filipin, a caveolae pathway inhibitor; Fig. 5G, chlorpromazine, an inhibitor of the clathrin pathway). These results further confirmed that PTK7-GEMs-cy5 were mainly taken up by 5637 cells via macropinocytosis. Further, the same endocytosis pathway was observed in another bladder cancer cell line T24 with PTK7 overexpression (Additional file 1: Fig. S16A -G). Meanwhile, we traced the intracellular events of PTK7-GEMs in bladder cancer cell lines 5637 and T24, PTK7-GEMs-Cy5 was first co-localized with lysosomes after co-incubated with bladder cancer cells in 2 h, then followed by lysosomal escape at 4 h (Additional file 1: Fig. S17A and B), and the onset of apoptosis could be observed after 8 h (Additional file 1: Fig. S17C and D). In addition, we also studied the co-localization of PTK7-GEMs-cy5 with endocytosis markers in the SV-HUC-1 cell line (Additional file 1: Fig. S18). Scattered red dot fluorescence in cells was observed, which was not coincident with the green fluorescence markers of the three endocytosis pathways (Additional file 1: Fig. S18A-C). Therefore, PTK7-GEMs-cy5 to normal SV-HUC-1 cells is unlikely to involve explicit endocytosis; the most likely explanation was nonspecific attachment.

### Biological imaging and anti-tumor activity of PTK7-GEMs in xenotransplanted subcutaneous tumors

**Fig. 6:**
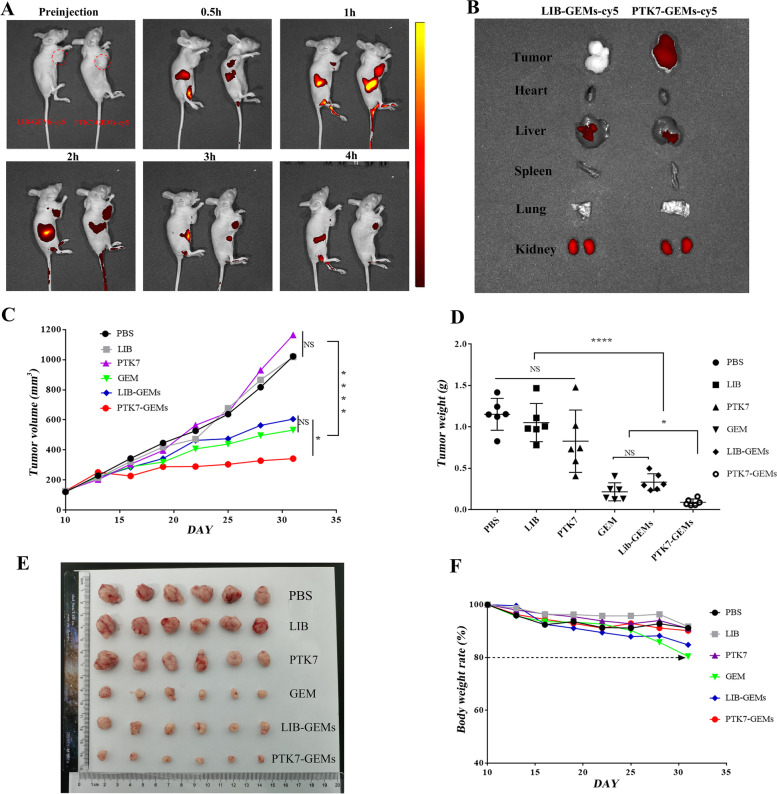
Optical imaging and Anti-tumor activity of PTK7-GEMs in xenotransplanted subcutaneous tumors. **A** Time-lapse fluorescence imaging at 0.5, 1, 2, 3, and 4 h after tail vein injection of Cy5-labeled PTK7-GEMs (right) or Lib-GEMs (left). The tumor site is indicated using a red circle. **B** Biodistribution of PTK7-GEMs-cy5 or Lib-GEMs-cy5 in the major viscera and tumors at 4 h after injection. **C** Growth curve of the tumors during therapy. In vivo evaluation of antitumor effects in the 5637 xenografted bladder cancer mouse model (*n* = 6 mice per group, *n* = 6 groups). **D** The tumor weights from the different groups are shown as a scattergram. **E** Images of tumor tissues from mice sacrificed at 32 days post inoculation. **F** Body weight changes during therapy. Data represents the mean ± SEM, *n* = 6; NS = not significant. *P < 0.05, **P < 0.01, ***P < 0.001, ****P < 0.0001

To verify the BC targeting of PTK7-GEMs-cy5, its tissue distribution was determined in a xenografted model of 5637 cells using in vivo imaging. In Fig. 6A, PTK7-GEMs-cy5 showed a strong fluorescence signal at 0.5 h after injection (righthand mice) than the control of LIB-GEMs-cy5, and accumulated rapidly, reaching a peak within 1 h, and could be retained at the tumor site for at least 4 h. Four hours later, after sacrificed, the tumors treated with PTK7-GEMs-cy5 showed much stronger fluorescence than that of LIB-GEMs-cy5 (Fig. 6B). PTK7-GEMs-cy5 and LIB-GEMs-cy5 were detected mainly in the liver and were excreted by the kidneys. These results showed that PTK7-GEMs has excellent tumor targeting in vivo. To further understand the anti-tumor effect of PTK7-GEMs in vivo, the efficacy of tumor growth inhibition by PTK7-GEMs was evaluated in a xenotransplanted subcutaneous tumor model using BC cell line 5637 (6 mice/each group). PTK7-GEMs had superior anticancer efficacy in terms of reduction in the tumor volume and tumor weight than GEM and LIB-GEMs treatment (Fig. 6C and D), while PBS, LIB and PTK7 did not show anti-tumor effects (Fig. 6E). Although CCK-8 and cell cycle experiments showed that GEM and PTK7-GEMs had similar cytotoxicity toward 5637 cells in vitro, PTK7-GEMs showed a better anti-tumor effect in vivo, which might be explained by better targeting of PTK7-GEMs in vivo. The malignant effect of the tumors resulted in weight loss in the GEM group being about 20%; however, in the PTK7-GEMs group only lost about 10% of their weight (Fig. 6F), with about 75% relative tumor growth inhibition, which was better than the GEM treatment group (55% tumor volume reduction) (Fig. 6C). These results demonstrated the advantages of using PTK7 aptamers as targeted drug delivery carriers reduced the systematical toxicity and increased the therapeutic efficacy of GEM. In addition, the PTK7-GEMs group displayed the weakest staining for the Ki67 antigen (brown) (Additional file 1: Fig. S19A), which indicates the greatest anti-tumor effect of PTK7-GEMs. The PTK7-GEMs group showed the brightest terminal deoxynulceotidyl transferase nick-end-labeling (TUNEL) signals (Additional file 1: Fig. S19B), indicating the induction of marked cancer cell apoptosis. Hematoxylin and eosin (H&E) staining of tumor tissue demonstrated the most obvious nuclear condensation and vacuolation in the PTK7-GEMs group (Additional file 1: Fig. S19C), suggesting higher levels of apoptosis and cell death.

### Anti-tumor activity of PTK7-GEMs in a lung metastasis model of BC

**Fig. 7:**
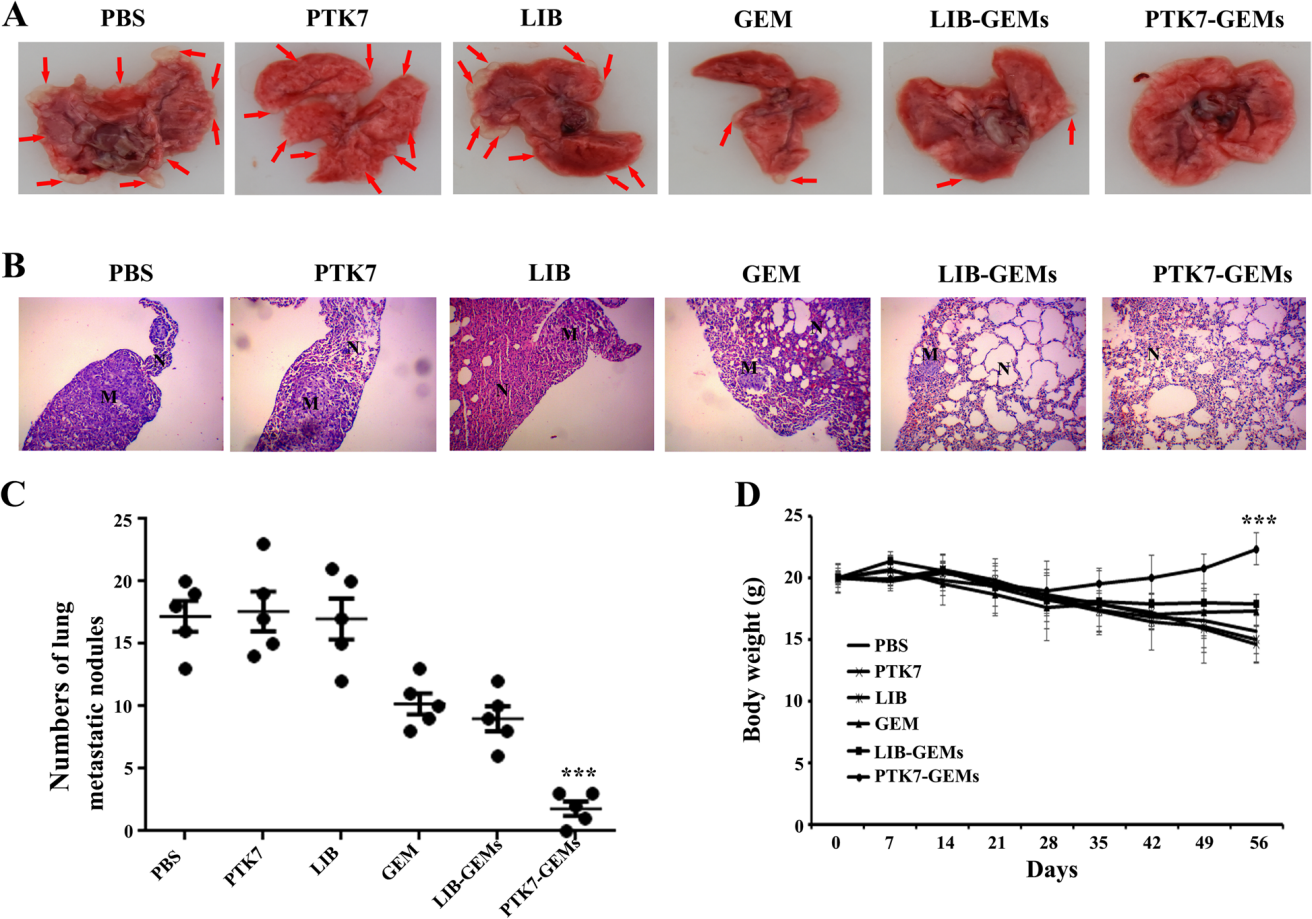
The antitumor activity of PTK7-GEMs in a mouse model of lung metastasis bladder cancer induced by tail vein injection. **A** Photographs of lungs excised from mice bearing bladder cancer cell tumors treated as indicated. **B** Photographs of lung tissue sections from mice bearing bladder cancer cell tumors under different treatments stained with H&E. **C** Comparison of pulmonary metastatic nodules of bladder cancer cells tumor-bearing mice treated as indicated. **D** Changes in the body weights of bladder cancer cells bearing mice under various treatments. All data are presented as the mean ± SD (*n* = 5). **P < 0.01, ***P < 0.001

A tail vein injection model of BC lung metastasis was used to assess PTK7-GEMs in vivo antitumor effect. The PTK7-GEMs-treated group displayed markedly lower levels of lung metastasis compared with that of the PBS group (control), PTK7 group and the LIB group (aptamer control). The PTK7-GEMs-treated group displayed an enhanced inhibitory effect against tumor metastasis compared with that of the GEM-treated or LIB-GEMstreated groups (Fig. 7A and B). There were significantly fewer pulmonary metastatic nodules in the PTK7-GEMs-treated group than in the GEM-treated group and the other groups (Fig. 7C). Notably, the mice treated with PTK7-GEMs showed no body weight loss (Fig. 7D), further verifying that PTK7-GEMs effectively inhibited tumor metastasis without any adverse effects. Contrastingly, the other groups experienced significant body weight loss, likely resulting from metastasisinduced lung malfunctions (Fig. 7D).

### Anti-tumor activity of PTK7-GEMs in an *in-situ* BC model

**Fig. 8:**
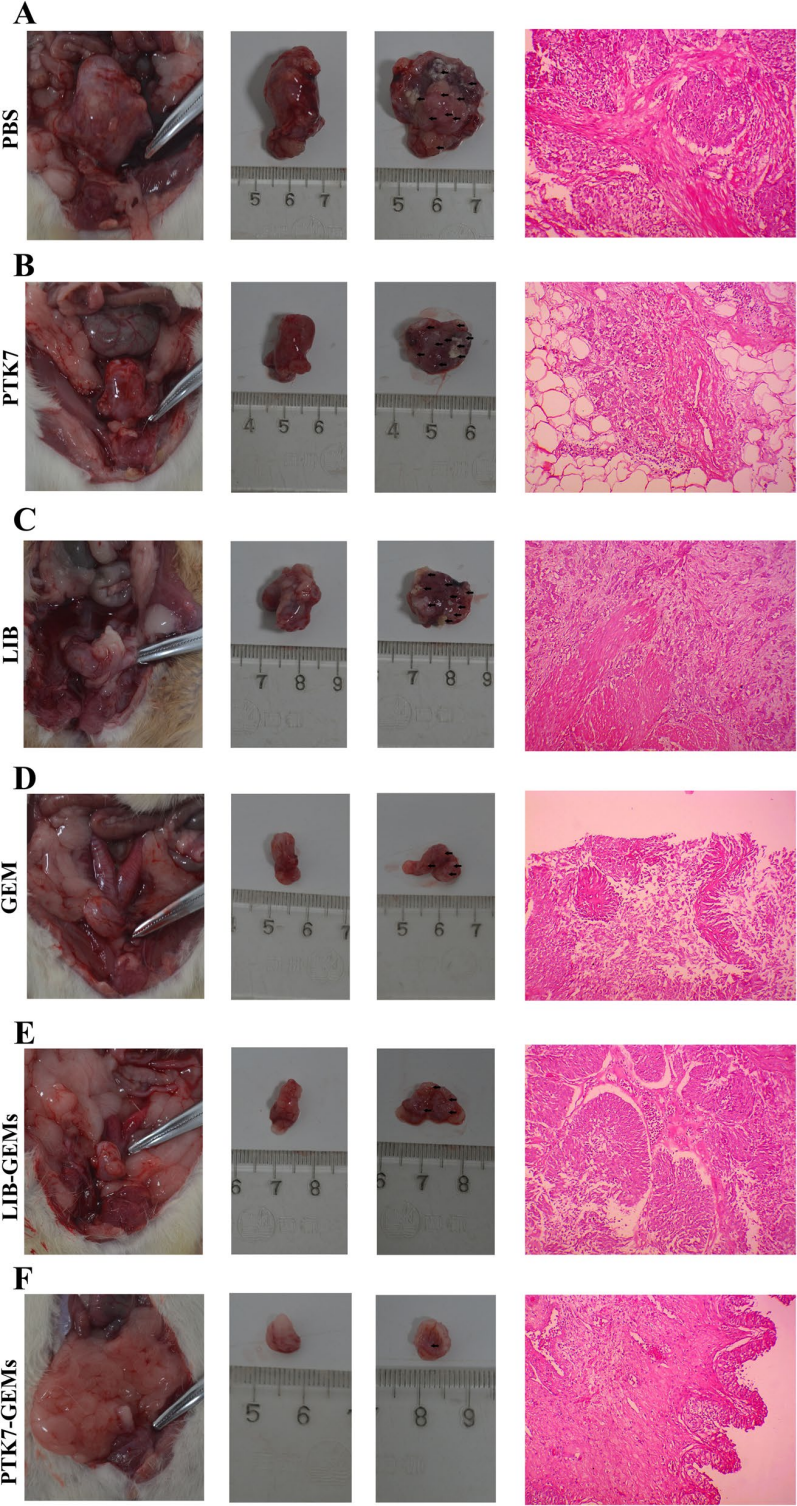
The antitumor activity of PTK7-GEMs in a model of in-situ bladder cancer model. **A** Photographs of H&E-stained tissue sections (muscle invasive bladder cancer at stage pT2 or higher and excised bladders after treatment with PBS. **B** Photographs of H&E-stained tissue sections (muscle invasive bladder cancer at stage pT2 or higher and excised bladders after treatment with PTK7. (**C**) Photographs of H&E-stained tissue sections (muscle invasive bladder cancer at stage pT2 or higher and excised bladders after treatment with LIB. **D** Photographs of H&E-stained tissue sections (noninvasive papillary carcinoma: stage pT1) and excised bladders after treatment with GEM. **E** Photographs of H&E-stained tissue sections (noninvasive papillary carcinoma: stage pT1) and excised bladders after treatment with LIB-GEMs. **F** Photographs of H&E-stained tissue sections (noninvasive papillary carcinoma: at stage pTa or lower) and excided bladders after treatment with PTK7-GEMs.

To treat BC, one of the main methods is urinary bladder instillation chemotherapy. Therefore, we further studied the clinical efficacy of PTK7-GEMs by determining whether it could suppress tumors in an *in-situ* model of BC. The weight of the rats’ bladder and the number of lesions were different among the treatment groups (Table S5). PTK7-GEMs treatment resulted in significant differences in the histopathology and bladder weight compared with those in the other treatment groups (*P* < 0.05). Moreover, PTK7-GEMs treatment resulted in lower tumor stage the majority of the examined bladder cancers (stage pT0/Ta/T1). By contrast, the tumors in the PBS and other control groups were mostly at stage pT2 or above. These results were verified by H&E staining of tissue sections and histological analysis of excised bladders (Fig. 8).

### The biosafety assessment of PTK7-GEMs in vivo

Considering the adverse side effects of GEM reported in clinic usage, whole blood and biochemical analysis were used to assess the toxicity of PTK7-GEMs in vivo [36, 37]. The contents of key enzymes were detected in blood, including liver function enzymes. Unsurprisingly, the alanine aminotransferase (ALT) and aspartate aminotransferase (AST) levels in the GEM and LIB-GEMs group were higher than PBS group, indicating hepatotoxicity. In contrast, that of PTK7-GEMs group were similar to those in the PBS treatment with no hepatotoxicity (Additional file 1: Table S6; Fig. S20). Meanwhile, the liver H&E staining analysis showed that both the GEM and LIBGEMs groups have varying degrees of hepatic cord loss, steatosis and sinusoid dilatation, while the PTK7-GEMs group showed a clear liver tissue structure with no obvious liver tissue damage (Additional file 1: Fig. S21A), Furthermore, no obvious structural injury in the main organs (Additional file 1: Fig. S21B - E) in all groups. Notably, no significant differences in creatine phosphokinase (CK), creatinine (CR), urea, white blood cells (WBC), hemoglobin (HGB), platelets (PLT), red blood cells (RBC), were observed in all treatment groups (Additional file 1: Table S6; Fig. S20 and S22). These results showed that no significant cardiotoxicity, nephrotoxicity, inflammation, granulocytopenia and anemia were observed in any treatment groups. Thus, PTK7GEM could overcome some side effects (e.g. hepatotoxicity) and showed good biosafety.

## Discussion

GEM is an effective first-line chemotherapeutical drug to treat BC, but it often leads to serious systemic toxicity and chemotherapy resistance because of its lack of recognition of tumor cells and have a low long-term response rate and high recurrence rate with side effects during the treatment of BC [38–40]. BC is sensitive to chemotherapeutics in the early stage; however, most patients develop long-term progression or recurrence. Recently, the effective treatment options for BC have increased considerably. Immunotherapy to treat metastatic BC comprises pembrolizumab, atezolizumab and ICIs in the second line setting, which have been approved for platinum–ineligible or patients who are not eligible for chemotherapy [41]. Furthermore, the ADC, enfortumab vedotin, comprises a fully human monoclonal antibody conjugated via a protease-cleavable linker to monomethyl auristatin E (MMAE), a clinically validated microtubule-disrupting agent, and is mainly available for metastatic BC [42]. Currently, immunotherapy regimens, targeted therapies, novel fibroblast growth factor receptors (FGFR) inhibitors, and ADCs are considered as promising treatment strategies for BC. However, in the changing field of therapeutic strategies for BC, the development and validation of biomarkers based on BC therapy are crucial.

The APDC strategy based on biomarkers is proposing as a magic bullet for anticancer drugs delivery. A previous study directly bound the PSMA specific aptamer A10 to doxorubicin, which interacts with the DNA double helix, and delivered it to the target cells [43]. PSMA overexpressing prostate cancer cells could be specifically destroyed after targeting by a gelonin-conjugated PSMA-specific aptamer A9 [44]. Various forms of chemical connection play an important role in APDCs [45–48]. To date, numerous APDCs have been approved in the clinic, in preclinical or clinical trials, such as Mucgen, Pegaptanib, RB006, Anticoagulant, ARC1779 and Nu172 [49–54]. Above all, routinely, aptamers display high specificity and affinity, and avoid protein-based immunogenicity, making them as excellent tools for drug delivery.

PTK7 high-expression has been reported in several cancers, and it has important signaling functions in the cancer development and progression [55, 56]. In this study, PTK7 was identified for the first time as a biomarker in BC through immunofluorescence assay in clinical samples and BC cell lines, whereas its mRNA and protein overexpression were not expressed on normal bladder uroepithelial cell line. The PTK7-targeted APDCs could directly bind to BC, and the GEM released from PTK7-GEMs could cause cancer cells collapse. The PTK7-GEMs might inhibit tumor growth and progression via multiple mechanisms. Firstly, PTK7-GEMs targeted transport reduces system consumption; Secondly, it solves the weak affinity of GEM toward BC, which results in more GEM accumulating in the tumor site; Thirdly, PTK7 might mediate more efficient pathways and reduce chemotherapeutic drug resistance; Lastly, the reduction in adverse side effects makes the body more tolerant to GEM and the long-term response rate to GEM might improve.

The novel targeting system, PTK7-GEMs, was established based on a biomarker (PTK7) in BC and through phosphodiester bond integrating three molecules of GEM onto one molecule of PTK7 aptamer, achieving high loading of anticancer drugs, and providing a scientific and reliable strategy to achieve better delivery capacity. The results showed that PTK7-GEMs were stable in serum and entered cells via macropinocytosis, then GEM was released from PTK7-GEMs under the action of intracellular phosphatase, which was consistent with that of the reported APDCs [57, 58]. To test their value in preclinical studies, three models of xenotransplantation (a subcutaneous tumor model, a lung metastatic tumor model, and an *in-situ* bladder cancer model) were used to verify the tumor targeting, anti-tumor efficacy, and biosafety of PTK7-GEMs [59]. Not surprisingly, PTK7-GEMs showed a satisfactory anticancer efficacy and could overcome the adverse side effects of GEM. All three models verified the anti-tumor efficacy and biosafety of PTK7-GEMs, which has important preclinical significance for the treatment of BC.

## Conclusion

In summary, we have identified PTK7 as a biomarker in BC, and synthesized a novel targeting system PTK7-GEMs based on auto-synthesis strategy. PTK7-GEMs specifically bind to BC dependent on the expression levels of PTK7. Furthermore, PTK7-GEMs showed stronger anti-tumor efficacy and excellent biosafety in three types of tumor xenograft mice models. These results demonstrated that PTK7-GEMs is a successful biomarker targeted APDCs strategy to treat BC. We believe this strategy using PTK7-GEMs will provide new directions for the precision treatment of BC in the field of biomarker-oriented tumor targeted therapy.

## Supplementary Information

**Additional file 1: Fig. S1.** The synthesis of GEM phosphoramidite 3. **Fig. S2.** The mass spectrum of GEM phosphoramidite 3. The calculated molecular weight was 950.03, the observed M/Z+ was 973.09 (M+Na+ = 973.09). **Fig. S3.** The 1H-NMR spectrum of GEM phosphoramidite 3. **Fig. S4.** The 13C-NMR spectrum of GEM phosphoramidite 3. **Fig. S5.** The step one of synthesis. The CpG linked with one of the bases A, G, C and T is selected as the solid phase carrier. **Fig. S6.** Formation of a phosphite bond. **Fig. S7.** Formation of a stable phosphodiester bond. **Fig. S8.** Acetyl blocking 5' OH is not involved in the reaction. **Fig. S9.** The synthesis of PTK7-GEMs. Synthesis proceeded from the 3' to 5 'end of the oligonucleotide, adding one base in each cycle. **Fig. S10.** The ESI-MS spectrum of PTK7-GEMs. **Fig. S11.** The ESI-MS spectrum LIB-GEMs. **Fig. S12.** The ESI-MS spectrum PTK7-1. **Fig. S13.** The ESI - MS of LIB-1. **Fig. S14.** The apoptosis analysis of 5637 and SV-HUC-1 cells. (A, B) The 5637 cells and SV-HUC-1 cells treated with LIB-GEMs, GEM or PTK7-GEMs respectively for 8 h and then incubated for 72 h with Complete medium before apoptosis analysis using flow cytometry. Upper right quadrant indicated advanced apoptotic cells. lower right quadrant indicated early apoptotic cells. **Fig. S15.** PTK7-GEMs cytotoxicity assays of bladder cancer cells. (A) T24 cell line treated with PTK7-GEMs, LIB-GEMs or GEM and evaluated by CCK8 assay. (B) IC50 of PTK7-GEMs and GEM against T24 cell line (NS, not significant). Data represents the mean ± SEM, *n* = 3. **Fig. S16.** PTK7-GEMs internalization and trafficking in bladder cancer cells. Confocal microscopy showing the co-localization of PTK7-GEMs-cy5 (red) with respective Alexa Fluor 488 labeled markers of endocytosis dextran (A), choleratoxin (B), transferrin (C). (D) Pearson’s correlation coefficient analysis of PTK7-GEMs-cy5 with endocytosis markers. Confocal microscopy revealing the co-localization of PTK7-GEMs-cy5 (red) with EIPA (E) (inhibitor of macropinocytosis), Filipin (F) (inhibitor of the caveolae pathway), and Chlorpromazine (G) (inhibitor of the clathrin pathway) (green). DAPI was used to counterstain the nuclei (blue). Scale bar, 10 μm. The means ± standard deviation (*n* = 3 per group) is indicated using error bars. Each replicate was from a single biological experiment and 10 independent fields of view were chosen from quantification. *P < 0.05; ns = not significant. **Fig. S17.** Escape of PTK7-GEMs from lysosomes in bladder cancer cells. Confocal microscopy images of 5637 (A) and T24 (B) cells co-incubated with lysotracker for 2 h and 4 h respectively. FAM (green) was used to stain endosomes/lysosomes, red was Cy5 labelled PTK7-GEMs, DAPI was used to stain nuclei (blue), and their merged images are showed. Apoptosis fate of 5637 (C) and T24 (D) cells after untreated or treated with PTK7-GEMs at 4 h or 8 h respectively, red indicates PI-stained dead or advanced apoptotic cells, red indicates Calcein-stained live healthy cells. **Fig. S18.** Internalization and trafficking of PTK7-GEMs in cells. Photographs show PTK7-GEMs-cy5 (red) co-localized with endocytic markers dextran (A), cholera toxin (B), and transferrin (C) labeled with Alexa Fluor 488 (green) respectively. The nuclei were counterstained with DAPI (blue). Scale bar, 10 μm. **Fig. S19.** Biosafety assessment of PTK7-GEMs in stained tumor sections. (A) Representative tumor sections stained for Ki67 (brown signal). (B) TUNEL staining (green fluorescence, merged with blue nuclei). (C) H&E staining in six groups. Scale = 50 μm. **Fig. S20.** Biosafety assessment of PTK7-GEMs in enzymes assays. Alanine aminotransferase (ALT), aspartate aminotransferase (AST), creatine phosphokinase (CK), Creatinine (CR), urea. Data are the mean ± SEM, *n* = 6; *P < 0.05 vs. the PBS group. **Fig. S21.** The biosafety assessment of PTK7-GEMs in staining of tissue sections. H&E staining analysis of A) Liver, B) Heart, C) Spleen, D) kidney and E) lung tissues from each group. Scale bars = 50 μm. “PBS” indicates PBS-treated xenografted mice. “LIB” indicates LIB-treated xenografted mice. “PTK7” indicates PTK7-treated xenografted mice. “GEM” indicates GEM-treated xenografted mice. “LIB-GEMs” indicates LIB-GEMs-treated xenografted. “PTK7-GEMs” indicates PTK7-GEMs-treated xenografted mice. **Fig. S22.** The biosafety assessment of PTK7-GEMs in biochemical assays. White blood cells (WBC), hemoglobin (HGB), platelets (PLT), red blood cells (RBC). Data are the mean ± SEM, *n* = 6; *P < 0.05 vs. the PBS group. **Table S1.** PTK7 expression and clinicopathological variables in bladder cancer. **Table S2.** Univariate cox proportional regression analysis for survival in bladder cancer. **Table S3.** Multivariate cox proportional regression analysis for survival in bladder cancer. **Table S4.** DNA sequences used in the study. **Table S5.** Tumor suppression effect of different treatment on bladder weight and histopathologic changes in SD rat bladders of different groups. **Table S6.** Hematological and biochemical data from sacrificed mice. 

## Data Availability

All the supplementary data in this study are available in additional file.

## Abbreviations

GEM: Gemcitabine
BC: Bladder cancer
MIBC: Muscle-invasive bladder cancer
NMIBC: Non-muscle-invasive bladder cancer
SMDs: Small molecular anticancer drugs
PTK7: Protein tyrosine kinase 7
PTK7-GEMs: Protein tyrosine kinase 7 aptamer-Gemcitabine conjugate
APDCs: Aptamer-drug conjugates
LIB-GEMs: Library free sequence aptamer-Gemcitabine conjugate
ssDNA: single-stranded Deoxyribonucleic acid
ssRNA: single-stranded Ribonucleic acid
SELEX: Systematic evolution of exponentially enriched ligands
ICIs: Immune checkpoint inhibitors
qRT-PCR: Quantitative real-time reverse transcription polymerase chain reaction
CCK-8: Cell counting kit-8
IF: Immunofluorescence staining
HPLC: High performance liquid chromatography
IHC: Immunohistochemistry
TEAA: Triethylamine acetate
FITC: Fluorescein isothiocyanate
NMS: Nude mice serum
FBS: Fetal bovine serum
BSA: Bovine serum albumin
ALT: Alanine aminotransferase
AST: Aspartate aminotransferase
CK: Creatine phosphokinase
CR: Creatinine
WBC: White blood cells
HGB: Hemoglobin
PLT: Platelets
RBC: Red blood cells.
