# Supplementary material for: Targeting treatment of bladder cancer using PTK7 aptamer-gemcitabine conjugate
Source: Biomater Res. 2022 Dec 5;26:74. doi: 10.1186/s40824-022-00328-9 (PMC9721011; doi:10.1186/s40824-022-00328-9)
Supplement: Supplementary file 1 — Additional file 1: Fig. S1. The synthesis of GEM phosphoramidite 3. Fig. S2. The mass spectrum of GEM phosphoramidite 3. The calculated molecular weight was 950.03, the observed M/Z+ was 973.09 (M+Na+ = 973.09). Fig. S3. The 1H-NMR spectrum of GEM phosphoramidite 3. Fig. S4. The 13C-NMR spectrum of GEM phosphoramidite 3. Fig. S5. The step one of synthesis. The CpG linked with one of the bases A, G, C and T is selected as the solid phase carrier. Fig. S6. Formation of a phosphite bond. Fig. S7. Formation of a stable phosphodiester bond. Fig. S8. Acetyl blocking 5' OH is not involved in the reaction. Fig. S9. The synthesis of PTK7-GEMs. Synthesis proceeded from the 3' to 5 'end of the oligonucleotide, adding one base in each cycle. Fig. S10. The ESI-MS spectrum of PTK7-GEMs. Fig. S11. The ESI-MS spectrum LIB-GEMs. Fig. S12. The ESI-MS spectrum PTK7-1. Fig. S13. The ESI - MS of LIB-1. Fig. S14. The apoptosis analysis of 5637 and SV-HUC-1 cells. (A, B) The 5637 cells and SV-HUC-1 cells treated with LIB-GEMs, GEM or PTK7-GEMs respectively for 8 h and then incubated for 72 h with Complete medium before apoptosis analysis using flow cytometry. Upper right quadrant indicated advanced apoptotic cells. lower right quadrant indicated early apoptotic cells. Fig. S15. PTK7-GEMs cytotoxicity assays of bladder cancer cells. (A) T24 cell line treated with PTK7-GEMs, LIB-GEMs or GEM and evaluated by CCK8 assay. (B) IC50 of PTK7-GEMs and GEM against T24 cell line (NS, not significant). Data represents the mean ± SEM, n = 3. Fig. S16. PTK7-GEMs internalization and trafficking in bladder cancer cells. Confocal microscopy showing the co-localization of PTK7-GEMs-cy5 (red) with respective Alexa Fluor 488 labeled markers of endocytosis dextran (A), choleratoxin (B), transferrin (C). (D) Pearson’s correlation coefficient analysis of PTK7-GEMs-cy5 with endocytosis markers. Confocal microscopy revealing the co-localization of PTK7-GEMs-cy5 (red) with EIPA (E) (inhibitor of macropinocytosis), [file 40824_2022_328_MOESM1_ESM.docx]

Supporting Information for

**Targeting treatment of bladder cancer using PTK7 aptamer-gemcitabine conjugate**

Wei Xiang^1†^, Yongbo Peng^2†^, Hongliang Zeng^1,3†^, Chunping Yu^4,5†^, Qun Zhang^6^, Biao Liu^1^, Jiahao Liu^1^, Xing Hu^1^, Wensu Wei^4,5^, Minhua Deng^4,5^, Ning Wang^4,5^, Xuewen Liu^7^, Jianfei Xie^8^, Weibin Hou^1^, Jin Tang^1^, Zhi Long^1^, Long Wang^1^ and Jianye Liu^1*^

^1^Department of Urology, The Third Xiangya Hospital of Central South University, No.138, Tongzipo Road, Changsha, 410013, Hunan, China

^2^Chongqing Key Laboratory for Pharmaceutical Metabolism Research, College of Pharmacy, Chongqing Medical University, No.1, Yixueyuan Road, Chongqing, 400016, China.

^3^Institute of Chinese Materia Medica, Hunan Academy of Chinese Medicine, No.8, Yuehua Road, Changsha 410013, China

^4^Department of Urology, Sun Yat-sen University Cancer Center, No. 651, Dongfeng Road East, Guangzhou, 510060, Guangdong, China

^5^State Key Laboratory of Oncology in Southern China, Collaborative Innovation Center for Cancer Medicine, No. 651, Dongfeng Road East, Guangzhou, 510060, Guangdong, China

^6^Department of Radiotherapy, The First Affiliated Hospital of Sun Yat-sen University, 58 Zhongshan 2nd Road, Guangzhou, 510080, Guangdong, China

^7^Department of Onology, The Third Xiangya Hospital of Central South University, No.138, Tongzipo Road, Changsha, 410013, Hunan, China

^8^Department of Nursing, The Third Xiangya Hospital of Central South University, No.138, Tongzipo Road, Changsha, 410013, Hunan, China

^†^These authors contributed equally to this work.

^*^**Corresponding author:** Jianye Liu, Department of Urology, The Third Xiangya Hospital of Central South University, No.138, Tongzipo Road, Changsha, 410013, Hunan, China. Fax: +86 731 88618808. Tel: +86 731 88618828. E-mail: liujianye810@163.com

**
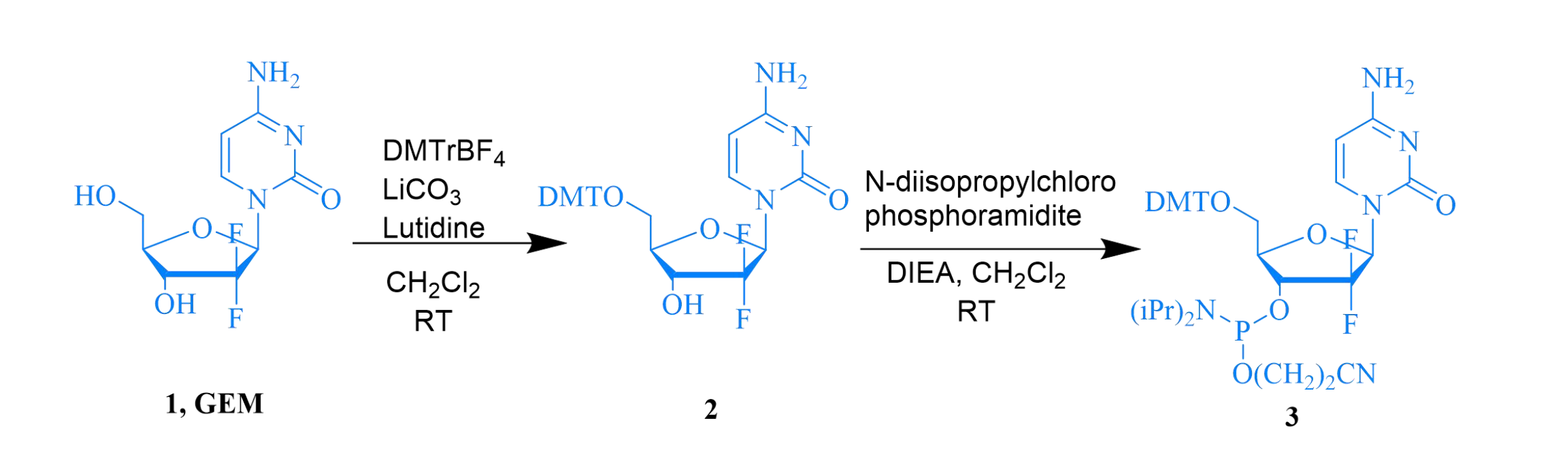
**

**Fig.** **S1** The synthesis of GEM phosphoramidite 3.


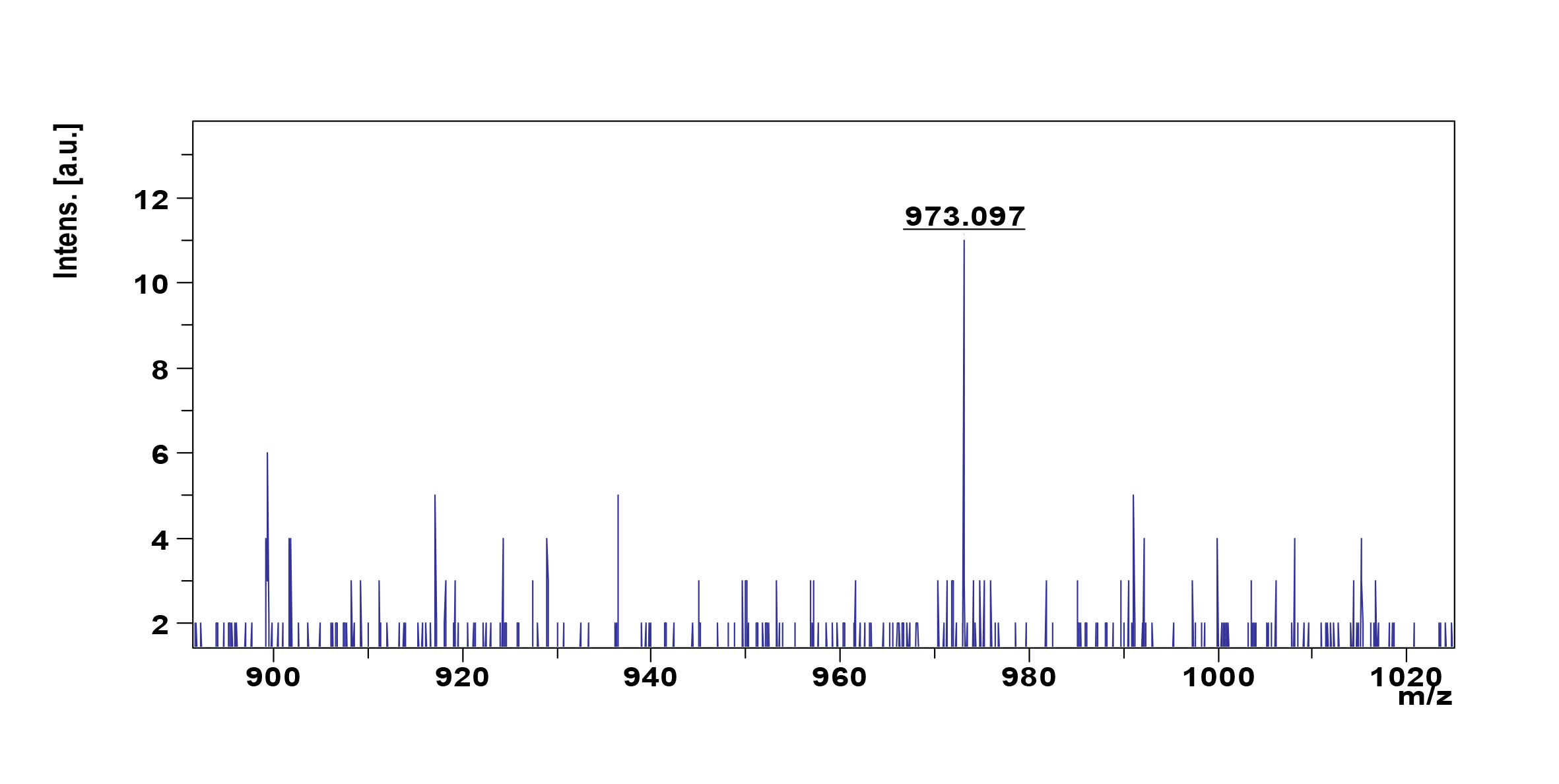


**Fig.** **S2** The mass spectrum of GEM phosphoramidite 3. The calculated molecular weight was 950.03, the observed M/Z+ was 973.09 (M+Na^+^ = 973.09).


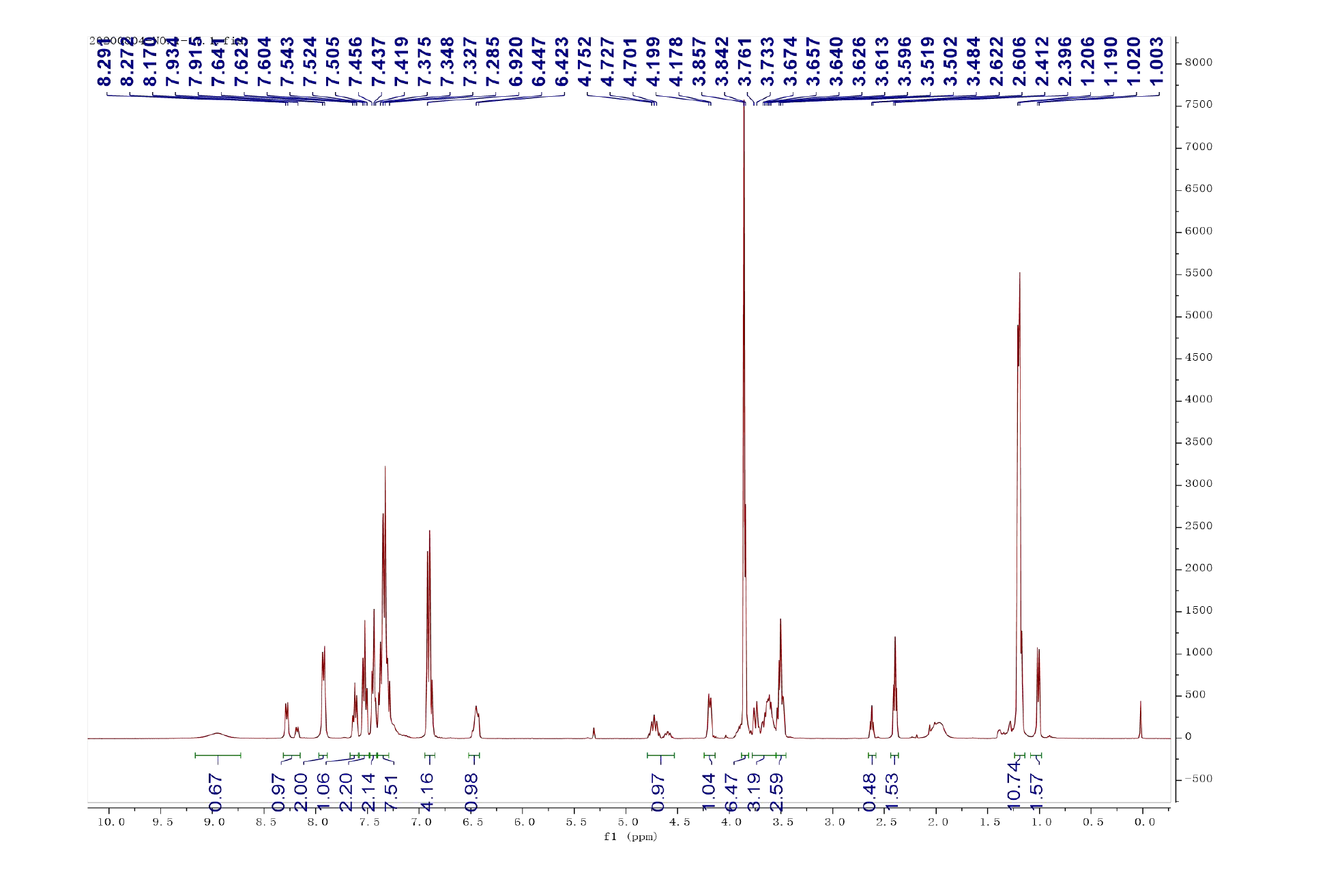


**Fig.** **S3** The ^1^H-NMR spectrum of GEM phosphoramidite 3.


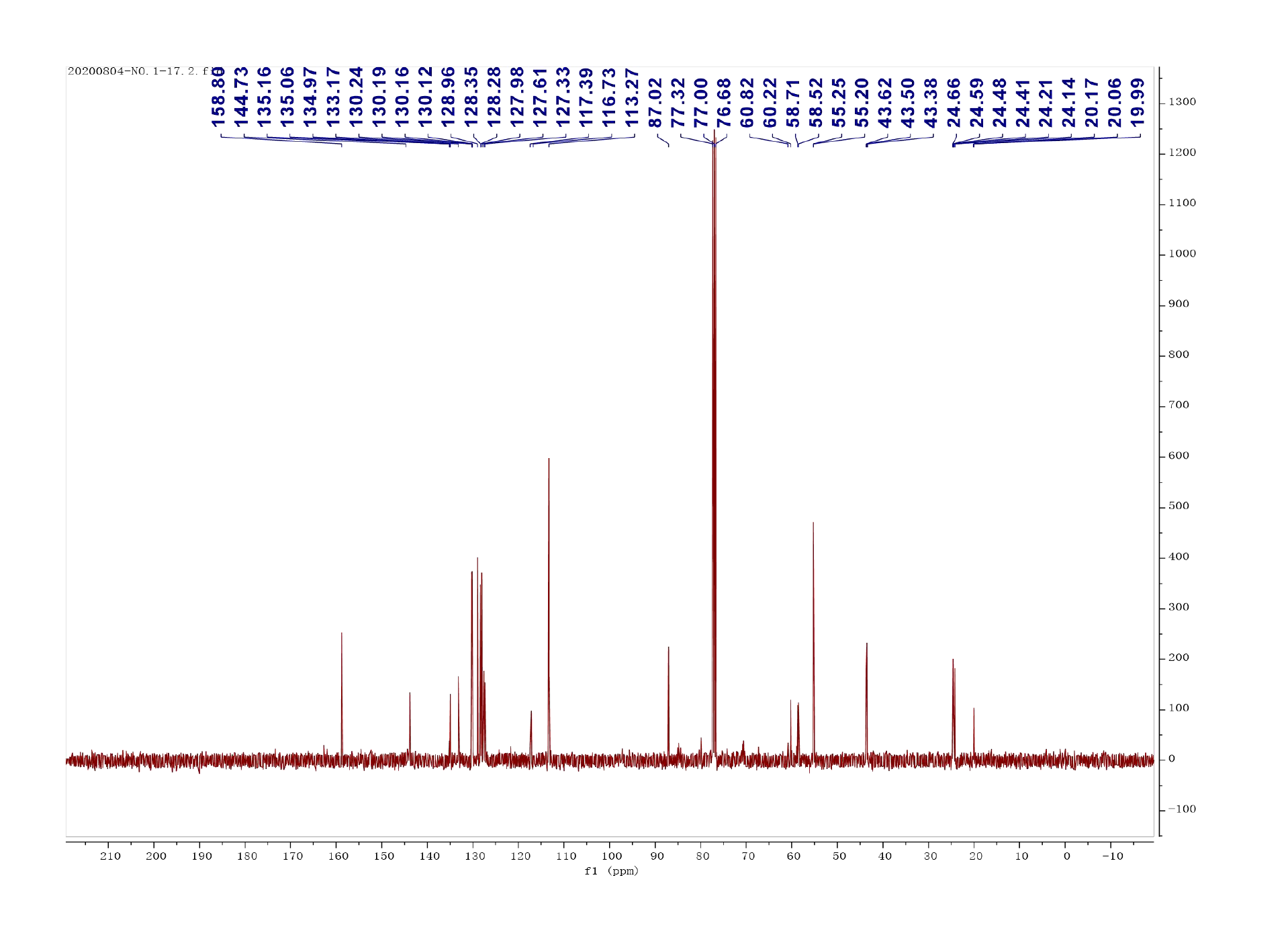


**Fig. S4** The ^13^C-NMR spectrum of GEM phosphoramidite 3.


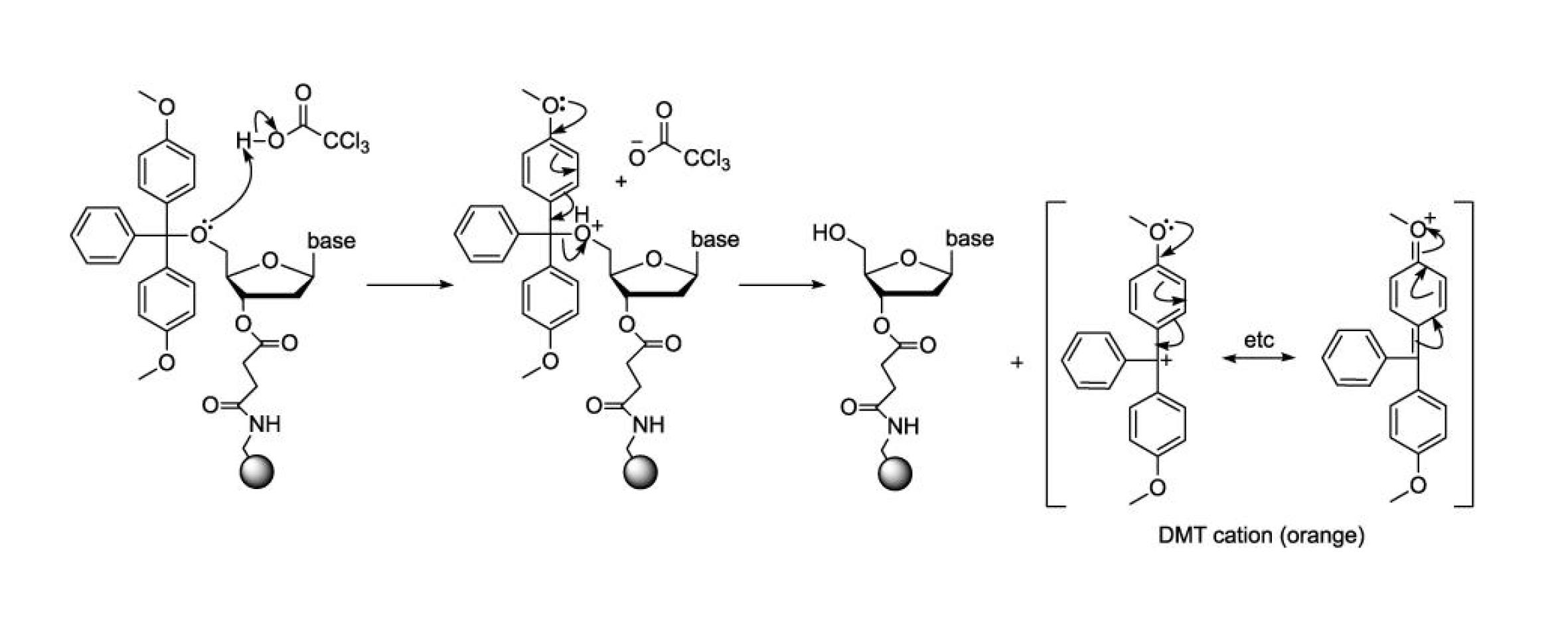


**Fig.** **S5** The step one of synthesis. The CpG linked with one of the bases A, G, C and T is selected as the solid phase carrier.


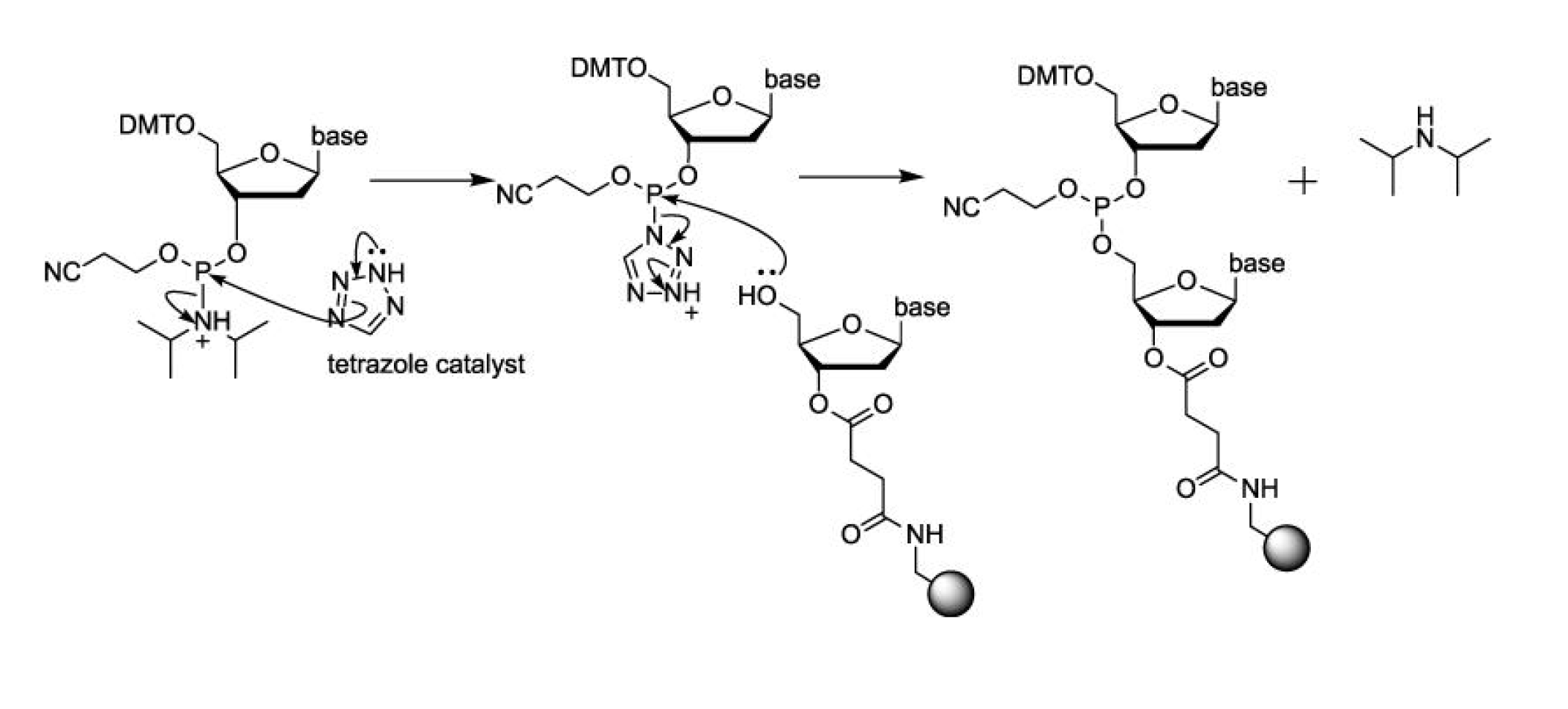


**Fig.** **S6** Formation of a phosphite bond.


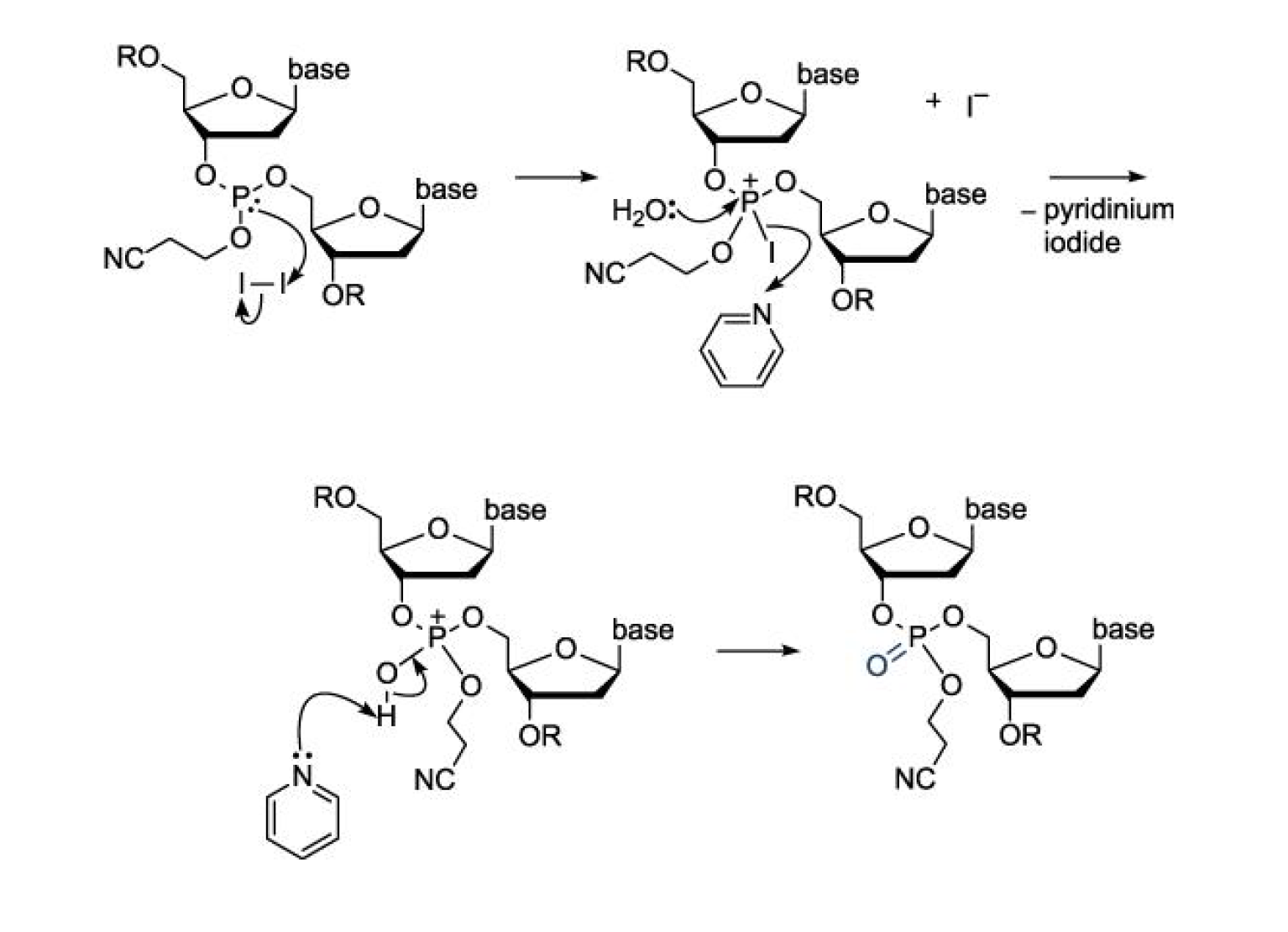


**Fig. S7** Formation of a stable phosphodiester bond.


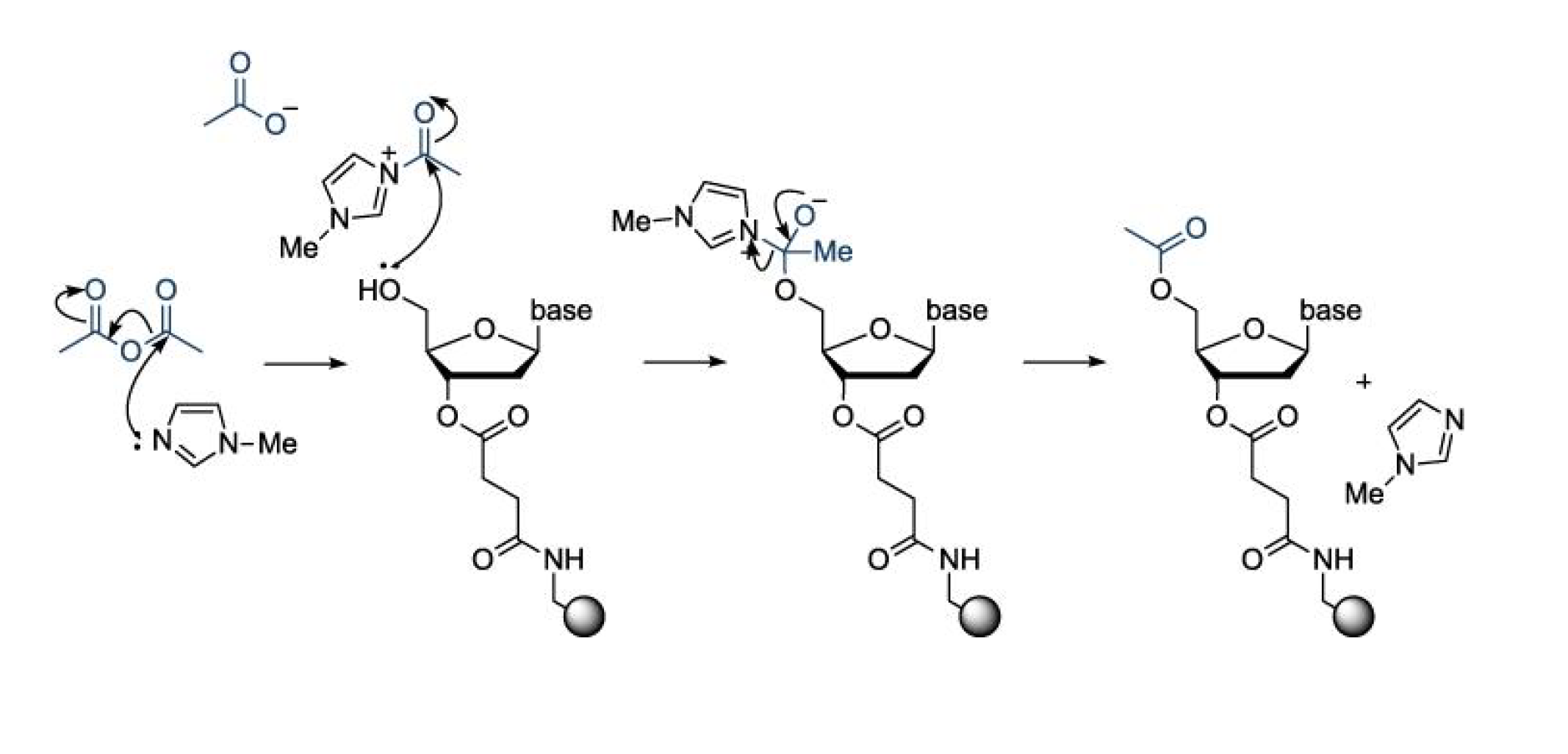


**Fig. S8** Acetyl blocking 5' OH is not involved in the reaction.


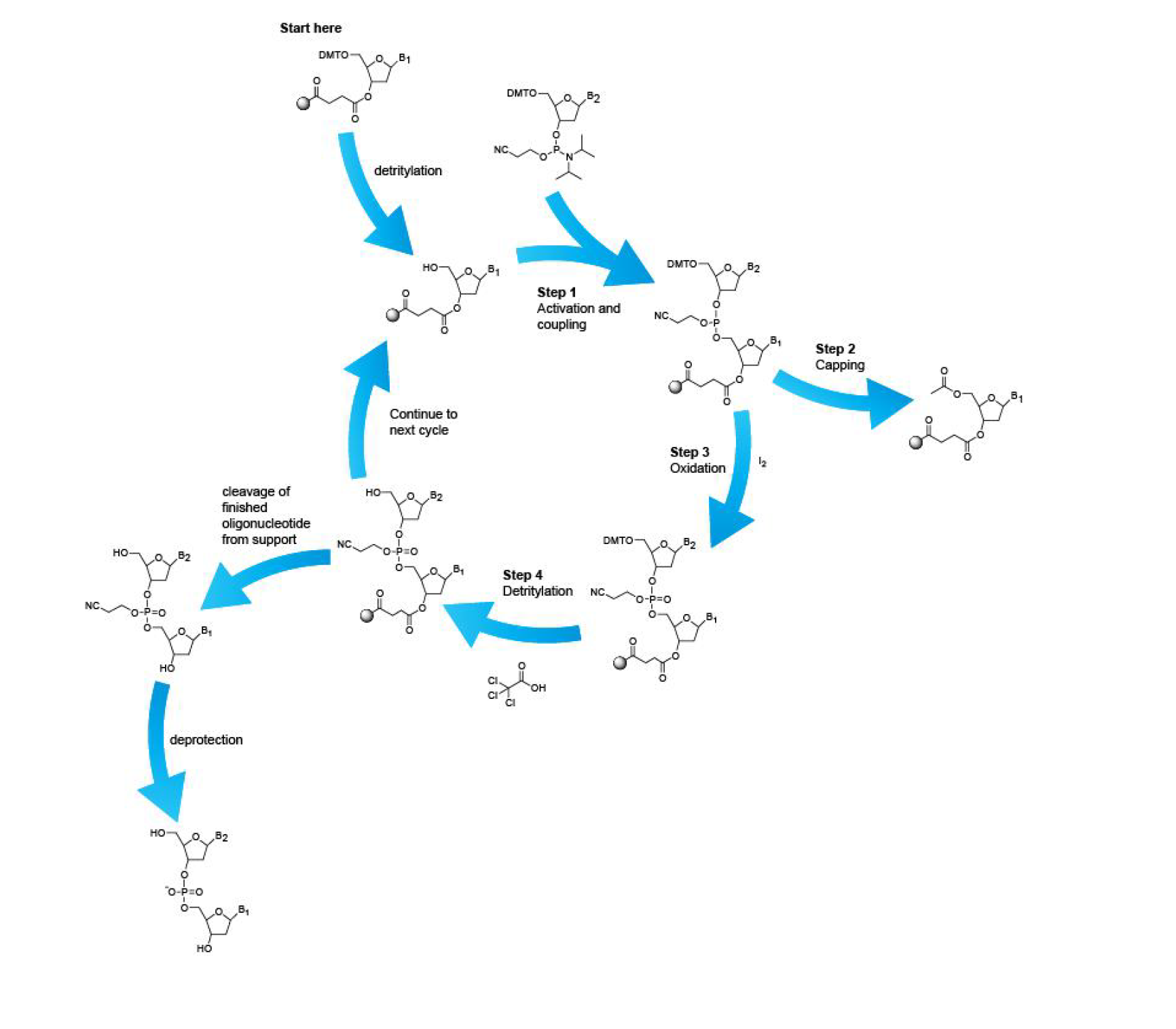


**Fig. S9** The synthesis of PTK7-GEMs. Synthesis proceeded from the 3' to 5 'end of the oligonucleotide, adding one base in each cycle.


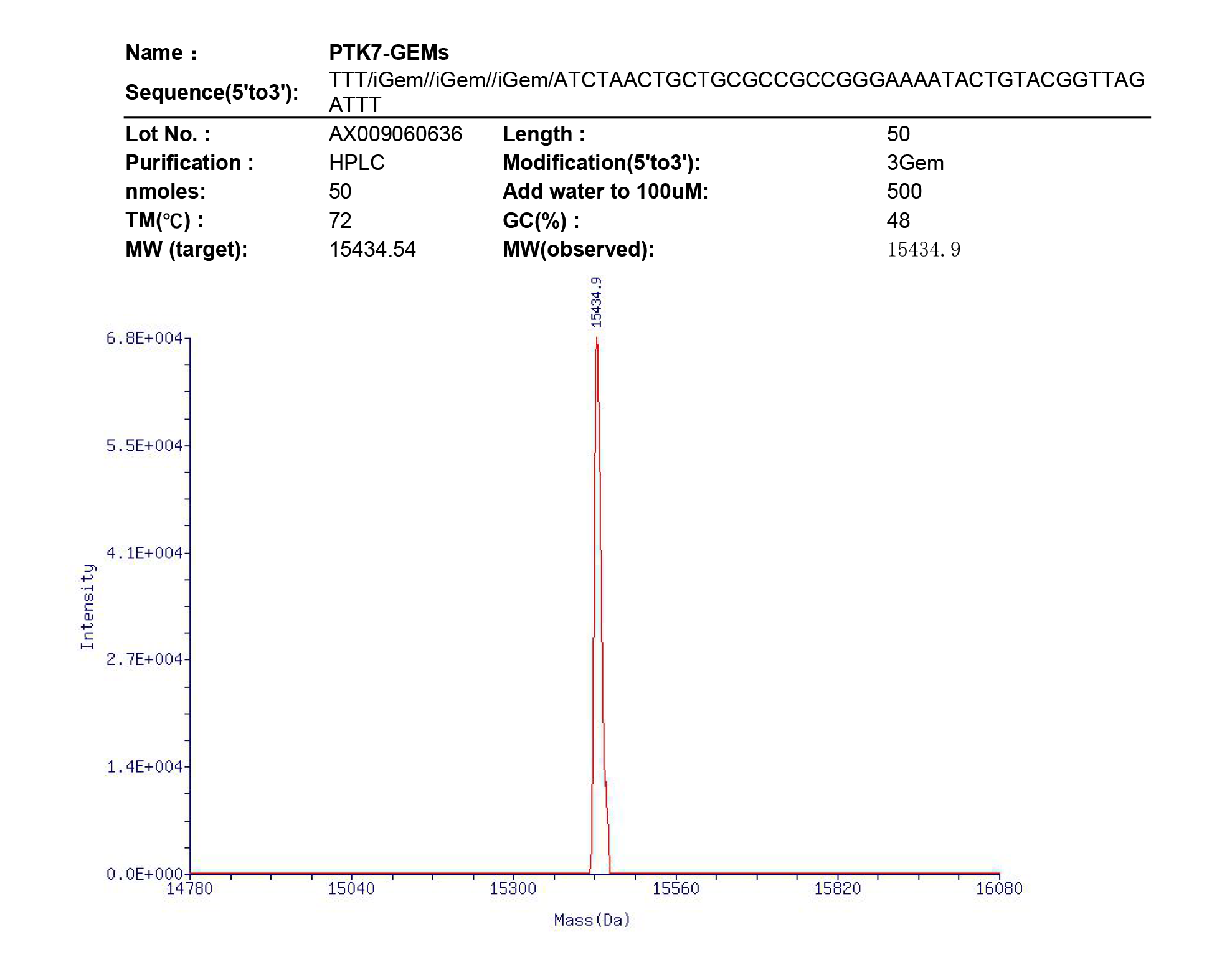


**Fig. S10** The ESI-MS spectrum of PTK7-GEMs.


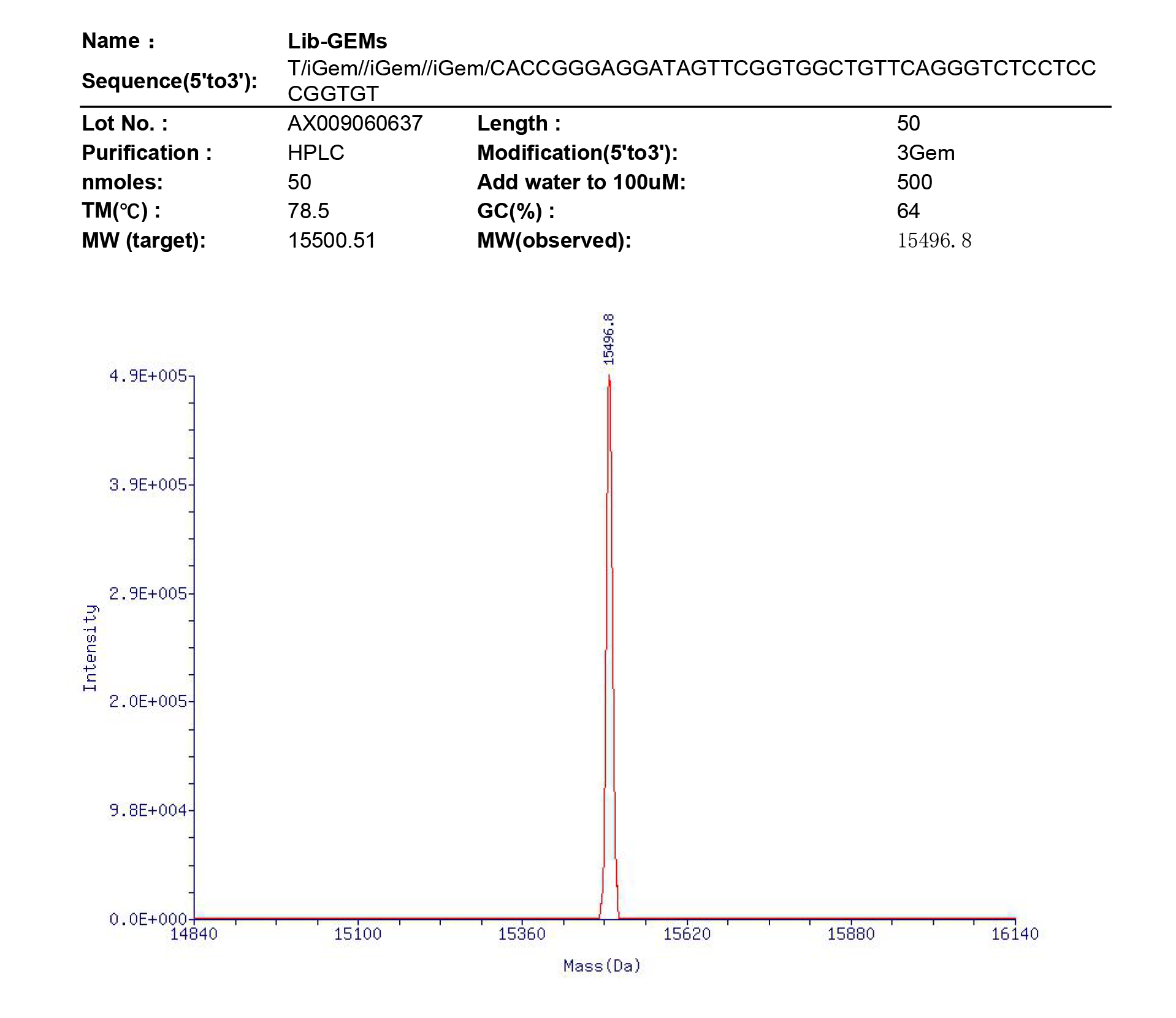


**Fig. S11** The ESI-MS spectrum LIB-GEMs.


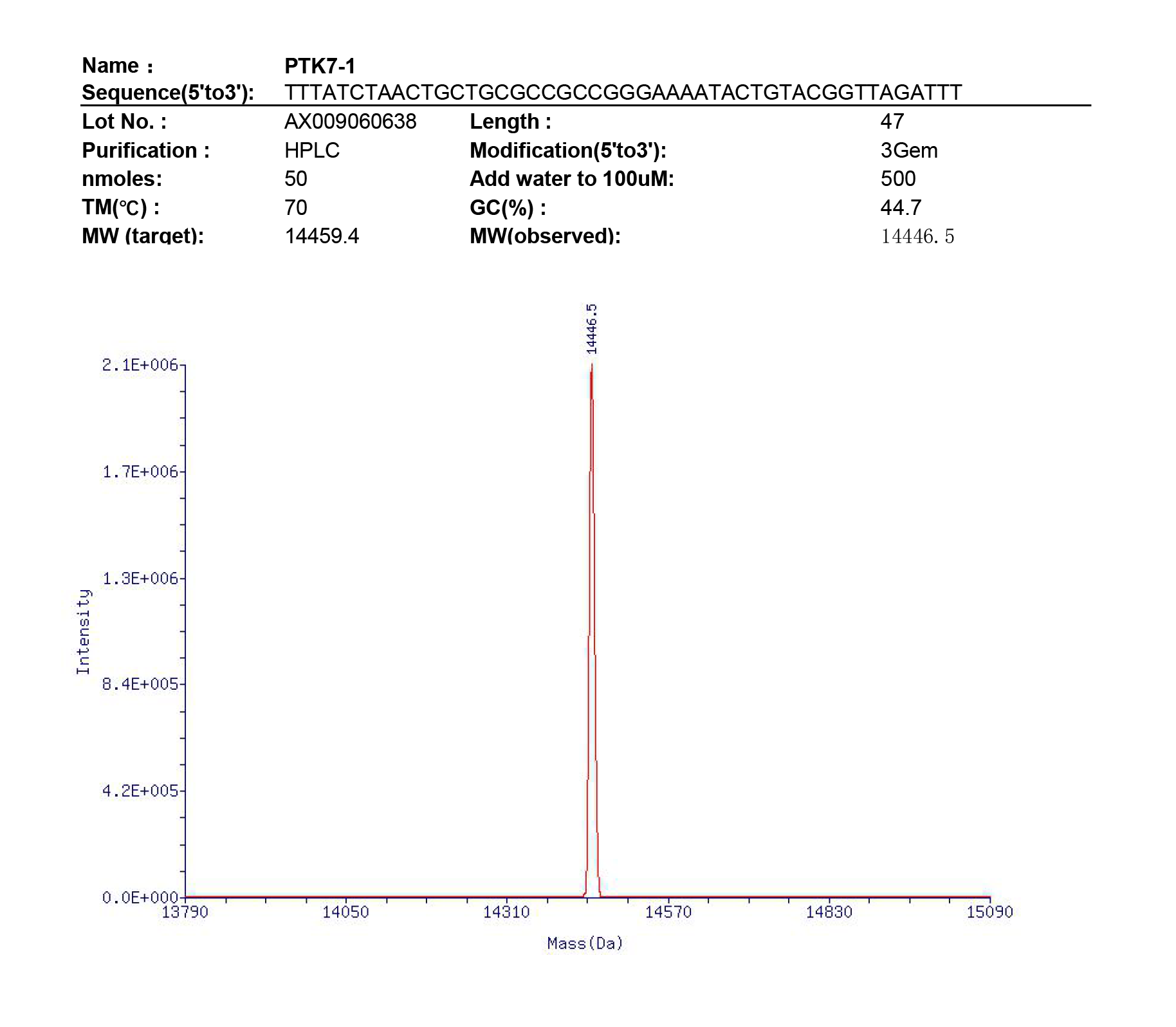


**Fig. S12** The ESI-MS spectrum PTK7-1.


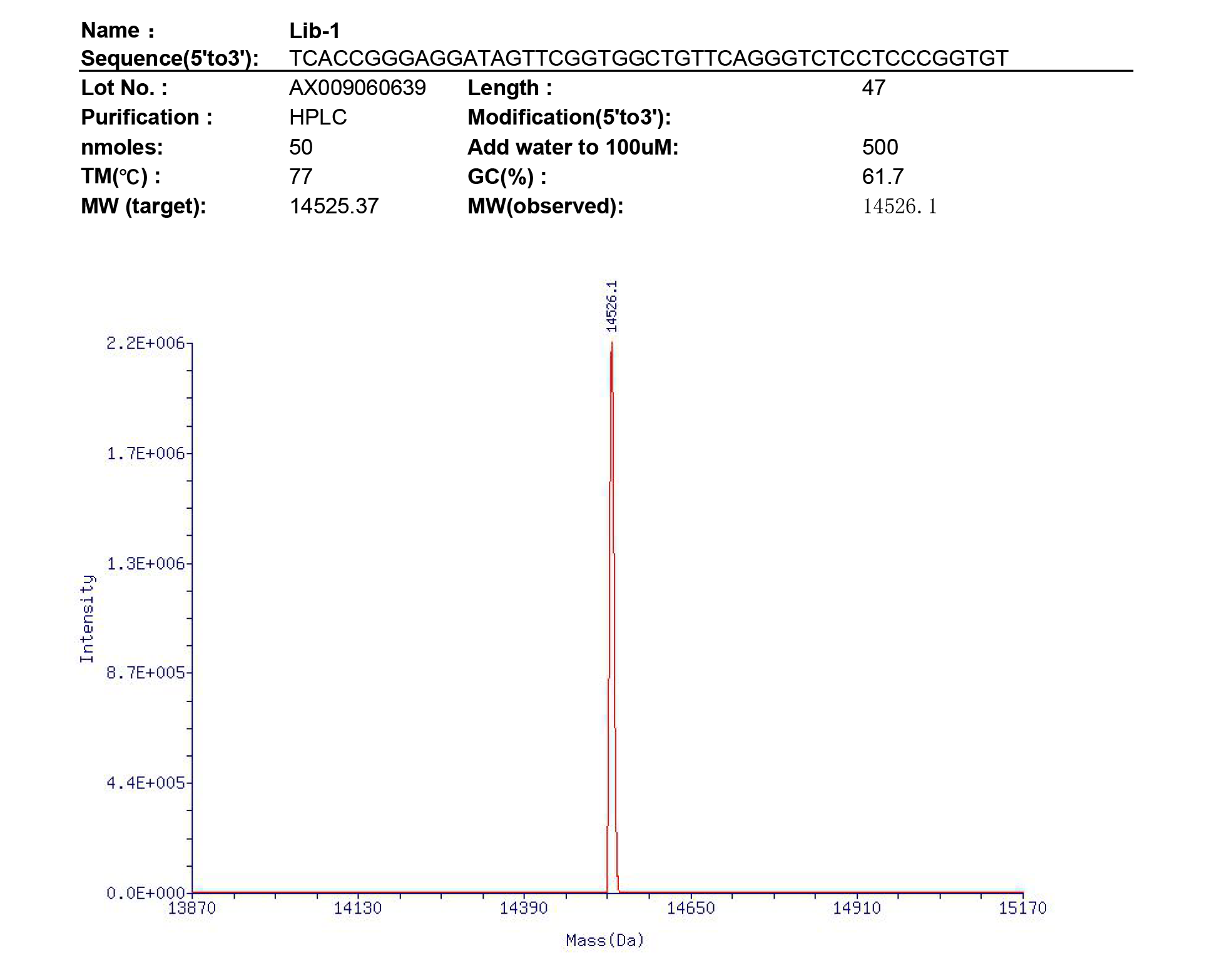


**Fig. S13** The ESI - MS of LIB-1.

**
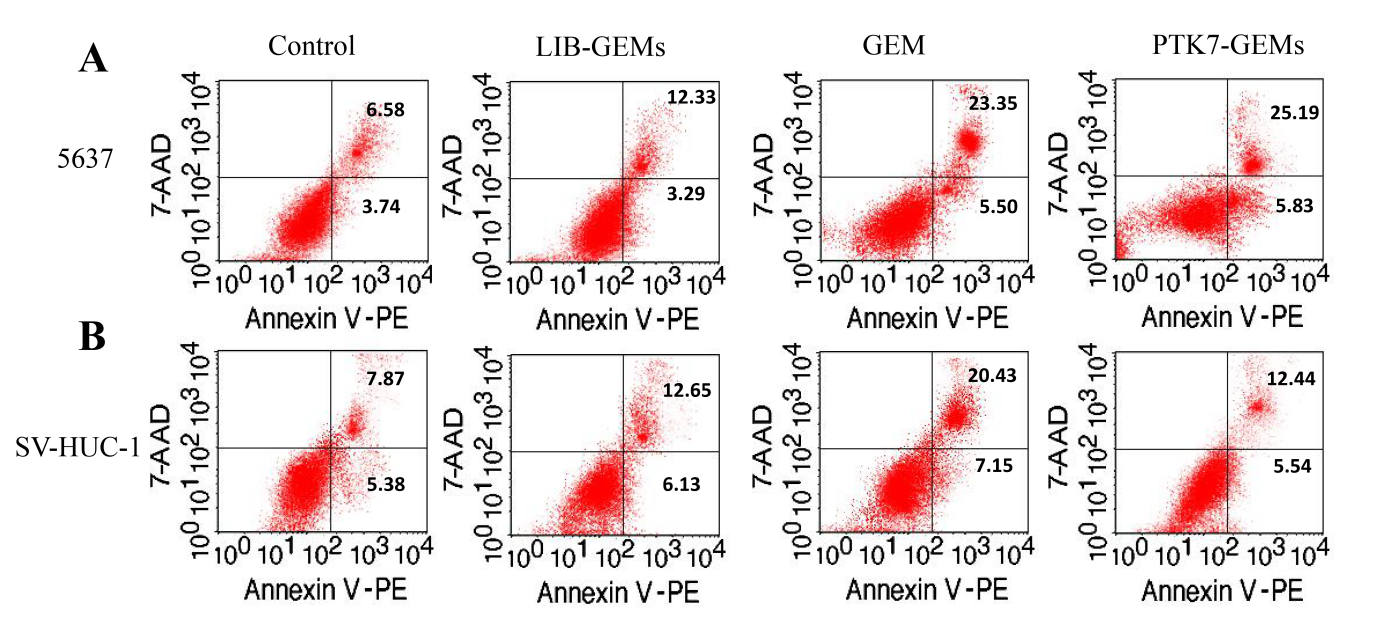
Fig. S14** The apoptosis analysis of 5637 and SV-HUC-1 cells. (**A, B**) The 5637 cells and SV-HUC-1 cells treated with LIB-GEMs, GEM or PTK7-GEMs respectively for 8 h and then incubated for 72 h with Complete medium before apoptosis analysis using flow cytometry. Upper right quadrant indicated advanced apoptotic cells. lower right quadrant indicated early apoptotic cells.


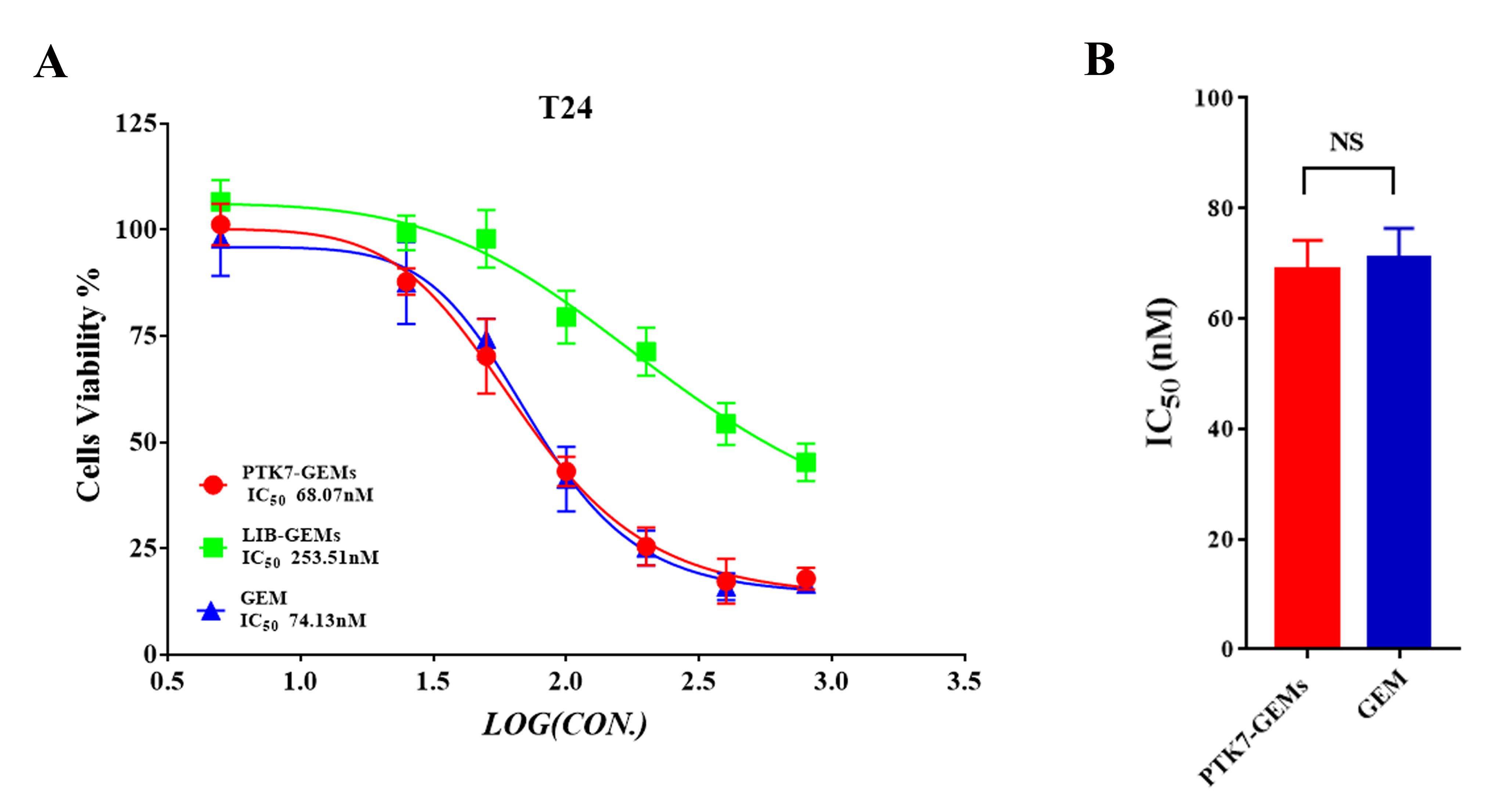


**Fig. S15** PTK7-GEMs cytotoxicity assays of bladder cancer cells. (**A**) T24 cell line treated with PTK7-GEMs, LIB-GEMs or GEM and evaluated by CCK8 assay. (**B**) IC_50_ of PTK7-GEMs and GEM against T24 cell line (NS, not significant). Data represents the mean ± SEM, n = 3.


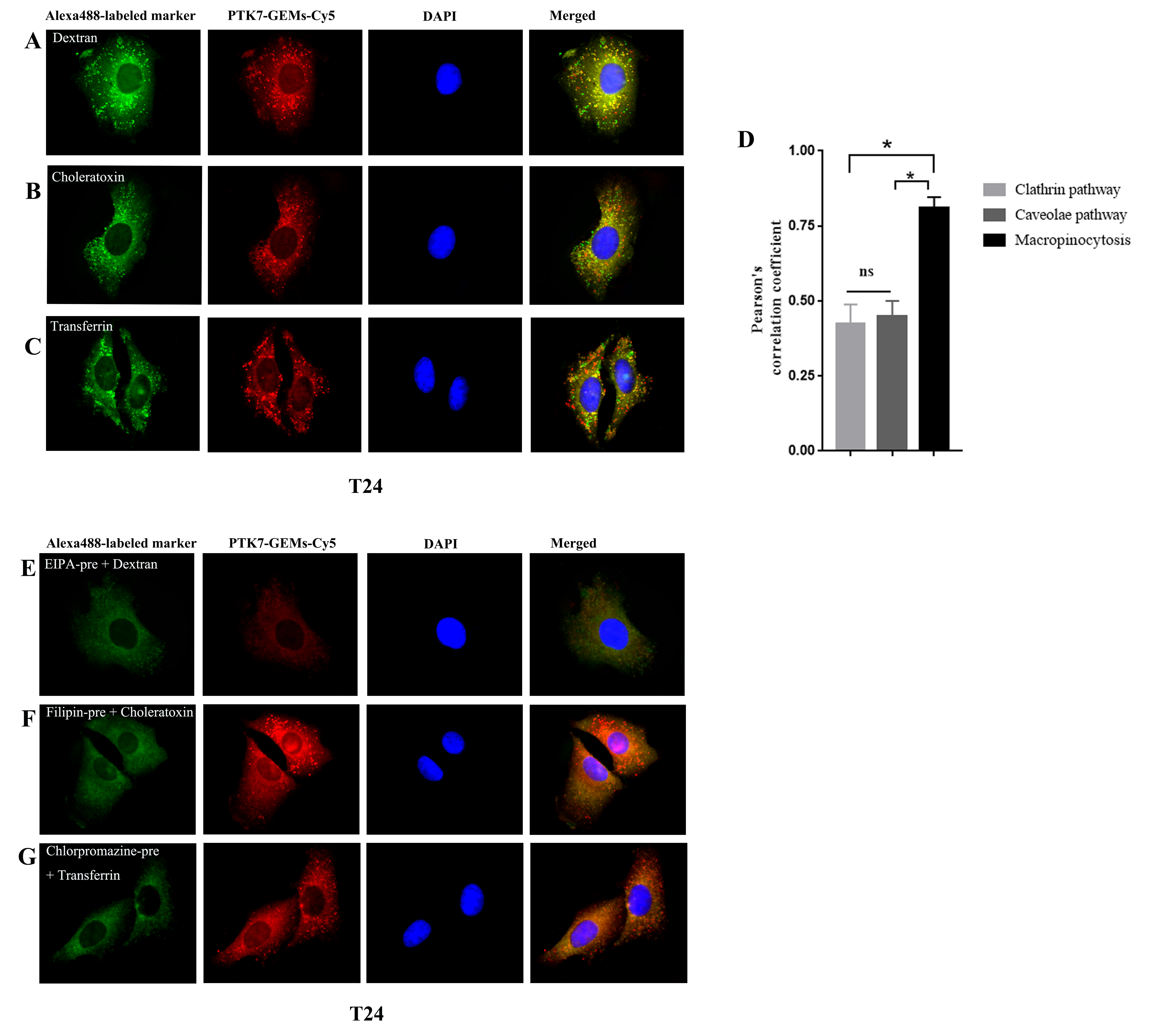


**Fig. S16** PTK7-GEMs internalization and trafficking in bladder cancer cells. Confocal microscopy showing the co-localization of PTK7-GEMs-cy5 (red) with respective Alexa Fluor 488 labeled markers of endocytosis dextran (**A**), choleratoxin (**B**), transferrin (**C**). (**D**) Pearson’s correlation coefficient analysis of PTK7-GEMs-cy5 with endocytosis markers. Confocal microscopy revealing the co-localization of PTK7-GEMs-cy5 (red) with EIPA **(E**) (inhibitor of macropinocytosis), Filipin (**F**) (inhibitor of the caveolae pathway), and Chlorpromazine (**G**) (inhibitor of the clathrin pathway) (green). DAPI was used to counterstain the nuclei (blue). Scale bar, 10 μm. The means ± standard deviation (n = 3 per group) is indicated using error bars. Each replicate was from a single biological experiment and 10 independent fields of view were chosen from quantification. **P* < 0.05; ns = not significant.


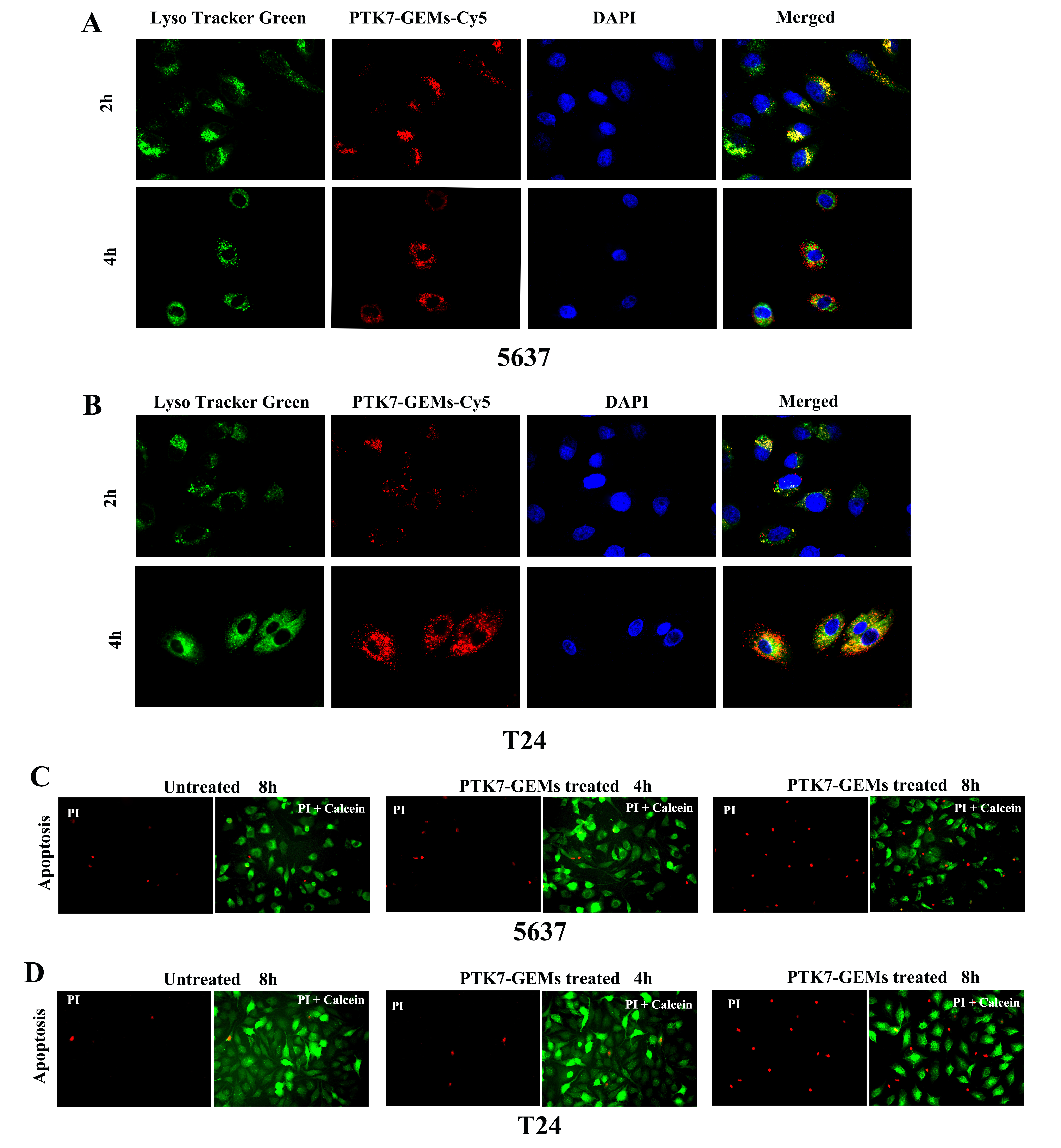


**Fig. S17** Escape of PTK7-GEMs from lysosomes in bladder cancer cells. Confocal microscopy images of 5637 (**A**) and T24 (**B**) cells co-incubated with lysotracker for 2 h and 4 h respectively. FAM (green) was used to stain endosomes/lysosomes, red was Cy5 labelled PTK7-GEMs, DAPI was used to stain nuclei (blue), and their merged images are showed. Apoptosis fate of 5637 (**C**) and T24 (**D**) cells after untreated or treated with PTK7-GEMs at 4 h or 8 h respectively, red indicates PI-stained dead or advanced apoptotic cells, red indicates Calcein-stained live healthy cells.

**
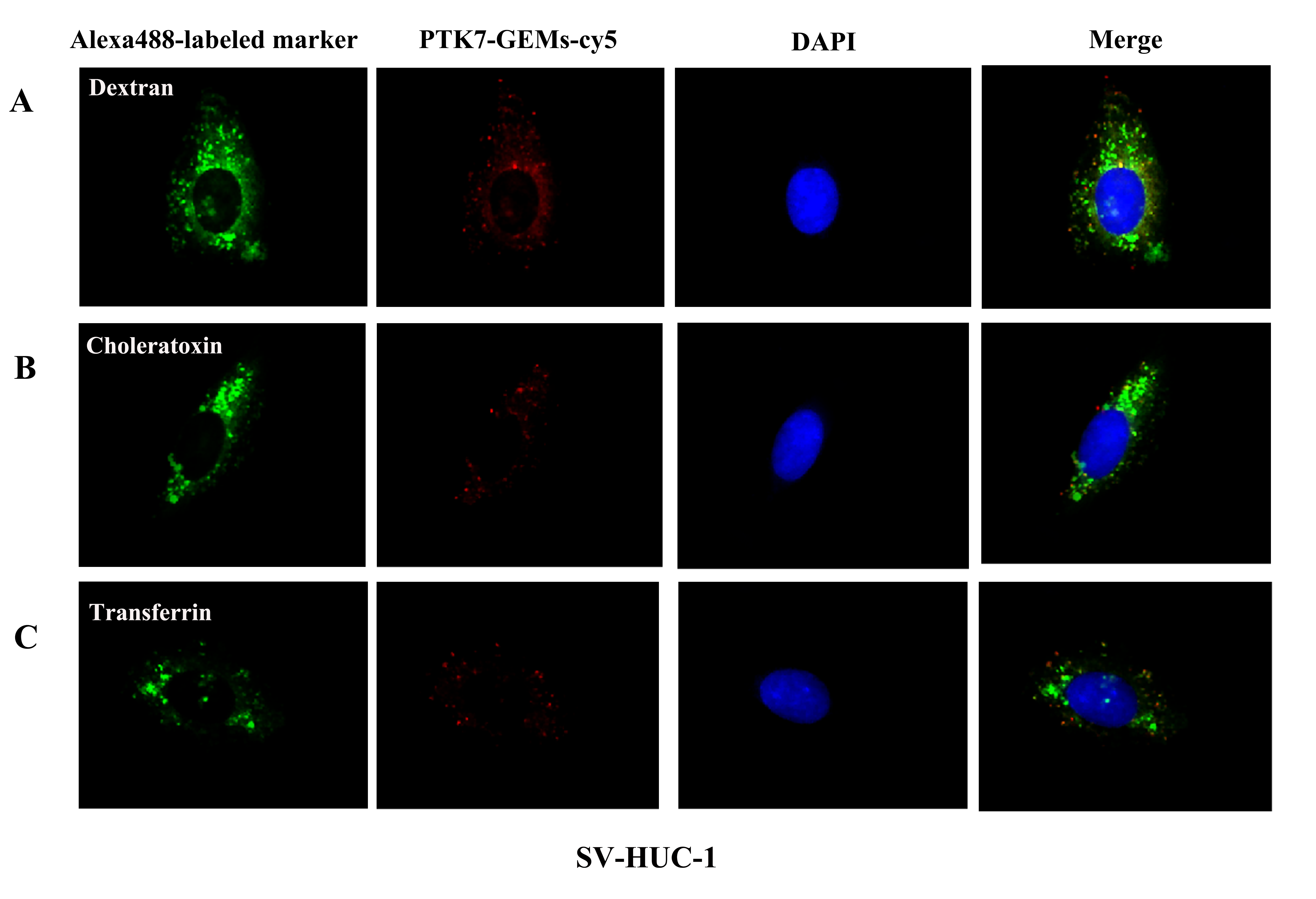
**

**Fig. S18** Internalization and trafficking of PTK7-GEMs in cells. Photographs show PTK7-GEMs-cy5 (red) co-localized with endocytic markers dextran (**A**), cholera toxin (**B**), and transferrin (**C**) labeled with Alexa Fluor 488 (green) respectively. The nuclei were counterstained with DAPI (blue). Scale bar, 10 μm.


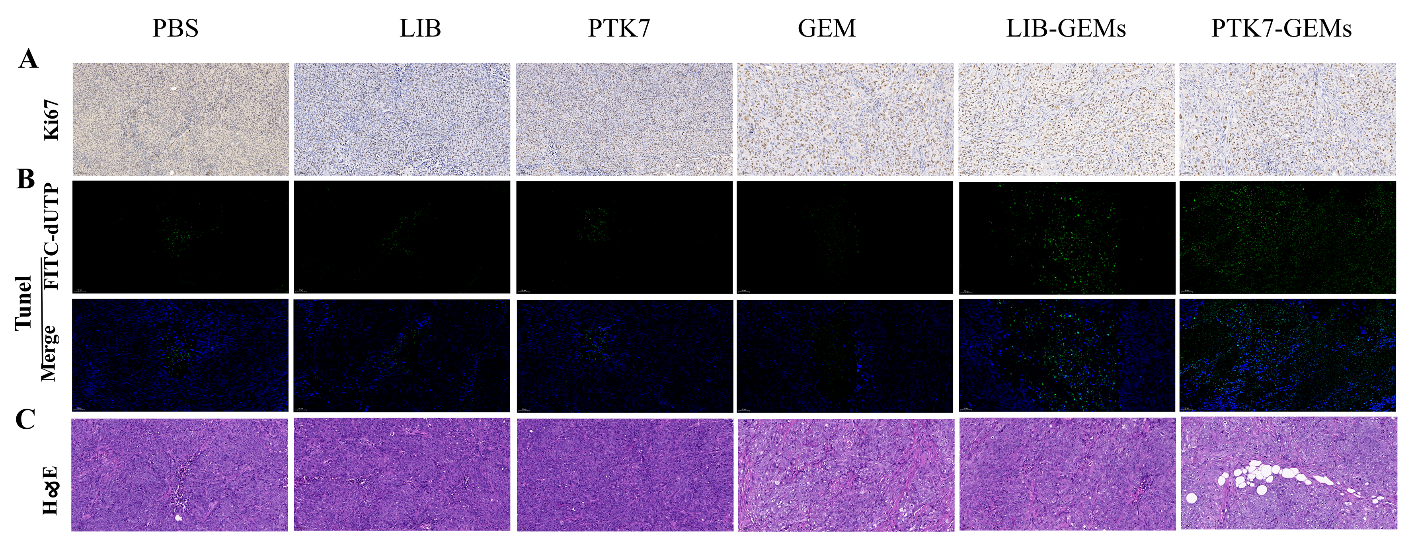


**Fig. S19** Biosafety assessment of PTK7-GEMs in stained tumor sections. **(A**) Representative tumor sections stained for Ki67 (brown signal). (**B**) TUNEL staining (green fluorescence, merged with blue nuclei). (**C**) H&E staining in six groups. Scale = 50 μm.


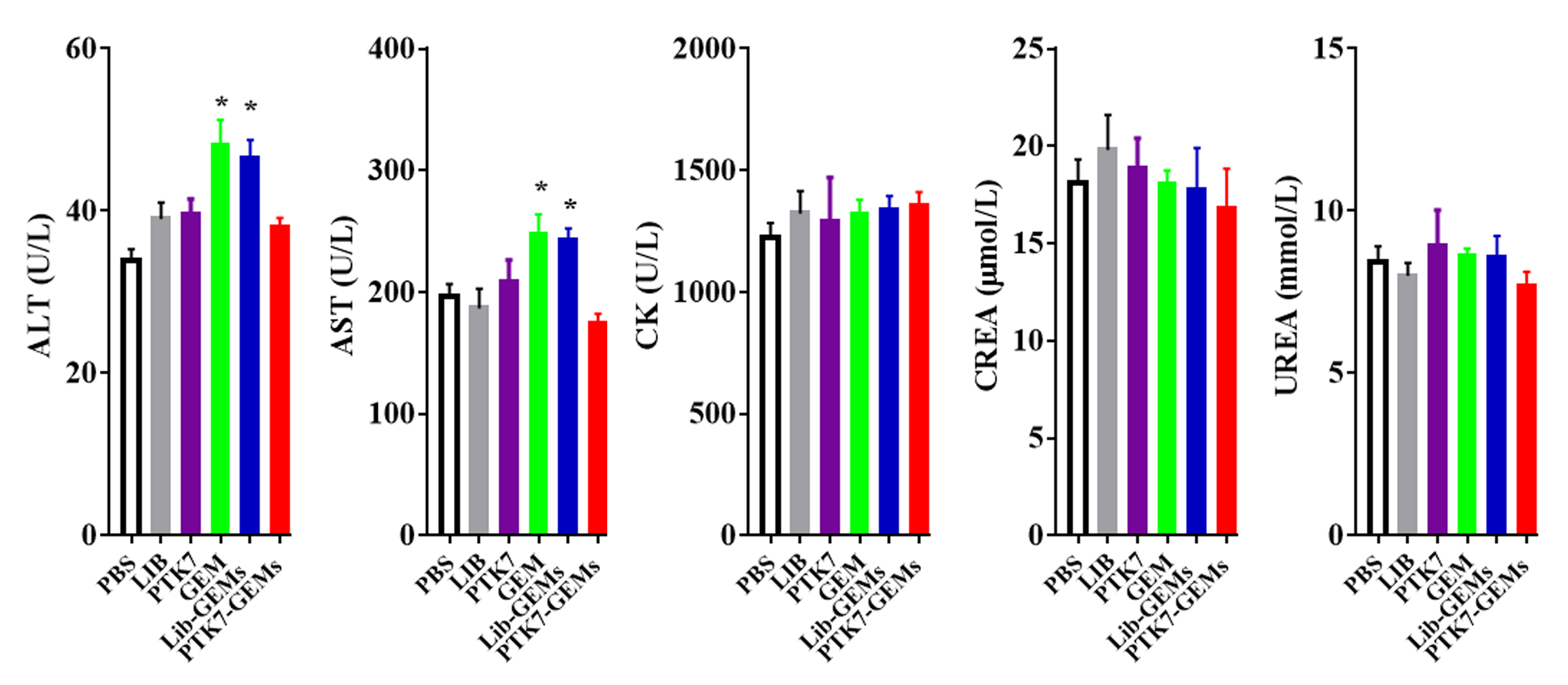


**Fig. S20** Biosafety assessment of PTK7-GEMs in enzymes assays. Alanine aminotransferase (ALT), aspartate aminotransferase (AST), creatine phosphokinase (CK), Creatinine (CR), urea. Data are the mean ± SEM, n = 6; **P* < 0.05 *vs*. the PBS group.


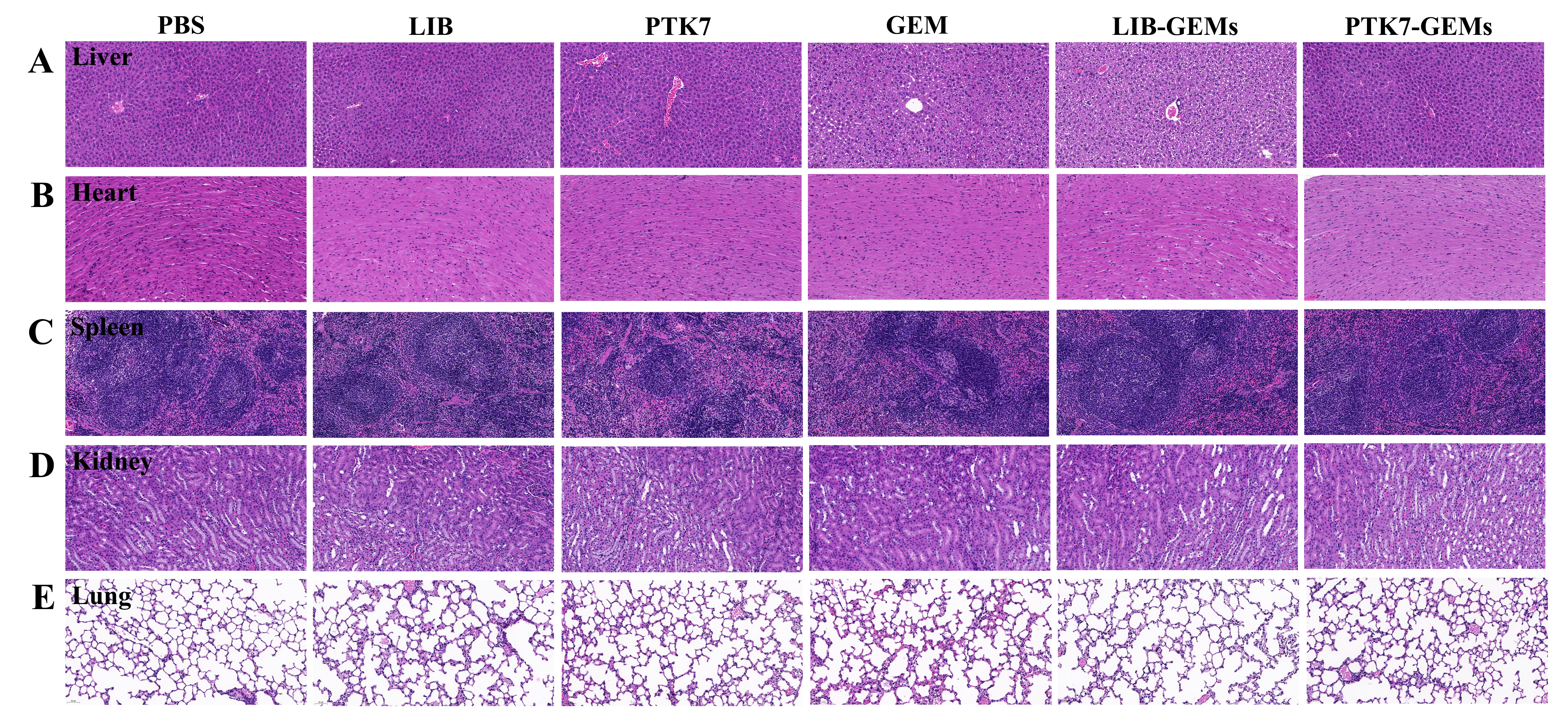


**Fig. S21** The biosafety assessment of PTK7-GEMs in staining of tissue sections. H&E staining analysis of **A**) Liver, **B**) Heart, **C**) Spleen, **D**) kidney and **E**) lung tissues from each group. Scale bars = 50 μm. “PBS” indicates PBS-treated xenografted mice. “LIB” indicates LIB-treated xenografted mice. “PTK7” indicates PTK7-treated xenografted mice. “GEM” indicates GEM-treated xenografted mice. “LIB-GEMs” indicates LIB-GEMs-treated xenografted. “PTK7-GEMs” indicates PTK7-GEMs-treated xenografted mice.


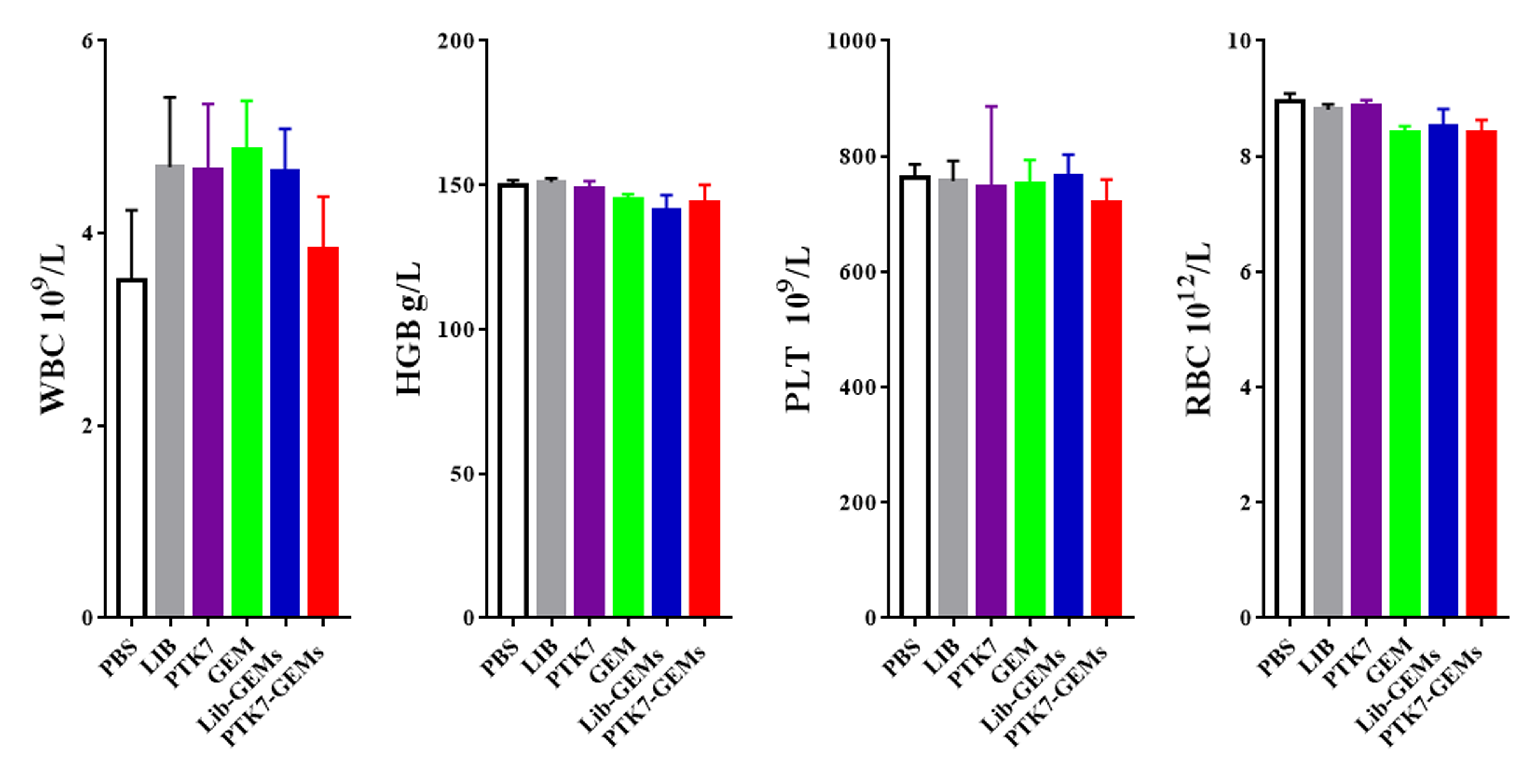


**Fig. S22** The biosafety assessment of PTK7-GEMs in biochemical assays. White blood cells (WBC), hemoglobin (HGB), platelets (PLT), red blood cells (RBC). Data are the mean ± SEM, n = 6; **P* < 0.05 *vs*. the PBS group.

| **Table S1.** PTK7 expression and clinicopathological variables in bladder cancer. | | | | |
| --- | --- | --- | --- | --- |
|  |  | PTK7 expression level | |  |
| Clinicopathological variables | Number of cases  (n = 148) | Low (%)  (n = 58) | High (%)  (n = 90) | P valueª |
| Age(years) |  |  |  | 0.561 |
| ≤60b | 62 | 26(41.9) | 36(58.1) |  |
| ＞60 | 86 | 32(37.2) | 54(62.8) |  |
| Gender |  |  |  | 0.638 |
| Male | 125 | 50(40.0) | 75(60.0) |  |
| Female | 23 | 8(34.8) | 15(65.2) |  |
| Tumor size(cm) |  |  |  | 0.713 |
| ≤3.8c | 84 | 34(40.5) | 50(59.5) |  |
| ＞3.8 | 64 | 24(37.5) | 40(62.5) |  |
| Tumor multiplicity |  |  |  | 0.914 |
| Unifocal | 39 | 15(38.5) | 24(61.5) |  |
| Multifocal | 109 | 43(39.4) | 66(60.6) |  |
| Tumor grade |  |  |  | 0.518 |
| Low | 59 | 25(42.4) | 34(57.6) |  |
| High | 89 | 33(37.1) | 56(62.9) |  |
| pT status |  |  |  | 0.018 |
| pT1 | 44 | 18(40.9) | 26(59.1) |  |
| pT2 | 52 | 27(51.9) | 25(48.1) |  |
| pT3/pT4 | 52 | 13(25.0) | 39(75.0) |  |
| pN status |  |  |  | 0.009 |
| pN- | 123 | 54(43.9) | 69(56.1) |  |
| pN+ | 25 | 4(16.0) | 21(84.0) |  |
| ^a^Chi-square test. bmean age. cmean size. | | | | |

**Table S2.** Univariate cox proportional regression analysis for survival in bladder cancer.

| Variables | All cases | HR (95% CI) | *P* valueª |
| --- | --- | --- | --- |
| Age(years) |  |  | 0.39 |
| ≤60^b^ | 62 | 1 |  |
| ＞60 | 86 | 1.288 (0.723-2.296) |  |
| Gender |  |  | 0.934 |
| Male | 125 | 1 |  |
| Female | 23 | 0.967 (0.435-2.150) |  |
| Tumor size(cm) |  |  | 0.596 |
| ≤3.8^c^ | 84 | 1 |  |
| ＞3.8 | 64 | 1.166 (0.661-2.055) |  |
| Tumor multiplicity |  |  | 0.998 |
| Unifocal | 39 | 1 |  |
| Multifocal | 109 | 1.001 (0.545-1.837) |  |
| Tumor grade |  |  | ＜0.001 |
| Low | 59 | 1 |  |
| High | 89 | 4.092 (1.978-8.466) |  |
| pT status |  |  | 0.013 |
| pT1 | 44 | 1 |  |
| pT2 | 52 | 2.029 (0.847-4.860) |  |
| pT3/pT4 | 52 | 3.365 (1.454-7.789) |  |
| pN status |  |  | ＜0.001 |
| pN- | 123 | 1 |  |
| pN+ | 25 | 3.878 (2.110-7.127) |  |
| PTK7 |  |  | 0.001 |
| Low | 69 | 1 |  |
| High | 79 | 3.082 (1.573-6.040) |  |

^a^Chi-square test. ^b^mean age. ^c^mean size.

**Table S3.** Multivariate cox proportional regression analysis for survival in bladder cancer.

| Variables | Hazards ratio | 95% CI^a^ | *P* value |
| --- | --- | --- | --- |
| Tumor grade (low vs. high) | 3.645 | 1.692-7.850 | 0.001 |
| pT status (pT1 vs. pT2 vs. pT3/pT4) | 1.415 | 0.947-2.114 | 0.036 |
| pN status (pN- vs. pN+) | 1.919 | 1.964-3.821 | 0.029 |
| PTK7 (low vs. high) | 3.105 | 1.561-6.177 | 0.001 |

^a^CI: confidence interval.

| **Table S4.** DNA sequences used in the study. | |
| --- | --- |
| Name | sequences and modification （5’-3’） |
| PTK7-GEMs-cy5 | TTT MMM ATC TAA CTG CTG CGC CGC CGG GAA AAT ACT GTA CGG TTA GAT TT (cy5) |
| LIB-GEMs-cy5 | TTT MMM NNN NNN NNN NNN NNN NNN NNN NNN NNN NNN NNN NNN NNN NNT TT (cy5) |
| PTK7-GEMs-FITC | TTT MMM ATC TAA CTG CTG CGC CGC CGG GAA AAT ACT GTA CGG TTA GAT TT (FITC) |
| LIB-GEMs-FITC | TTT MMM NNN NNN NNN NNN NNN NNN NNN NNN NNN NNN NNN NNN NNN NNT TT (FITC) |
| PTK7-GEMs | TTT MMM ATC TAA CTG CTG CGC CGC CGG GAA AAT ACT GTA CGG TTA GAT TT |
| LIB-GEMs | TTT MMM NNN NNN NNN NNN NNN NNN NNN NNN NNN NNN NNN NNN NNN NNT TT |
| PTK7 | TTT ATC TAA CTG CTG CGC CGC CGG GAA AAT ACT GTA CGG TTA GAT TT |
| LIB | TTT NNN NNN NNN NNN NNN NNN NNN NNN NNN NNN NNN NNN NNN NNT TT |
| Cy5-labeled and FITC-labeled DNA are modified on corresponding 3’ terminal. “MMM” stands for gemcitabine. “NNN” stands for free sequences. | |

| **Table S5.** Tumor suppression effect of different treatment on bladder weight and histopathologic changes in SD rat bladders of different groups. | | | | | | |
| --- | --- | --- | --- | --- | --- | --- |
| Group | No. of rats  sacrificed | Bladder weight (mg; Mean ± SD) | Normal (T0) | Cancer in situ or Noninvasive papillary carcinoma (Tis/Ta) | Subepithelial connective tissue invasive bladder cancer (T1) | Muscle invasive bladder cancer (≥ T2) |
| PBS control | 10 | 623±213 | 0 | 1 | 1 | 8 |
| PTK7 | 10 | 576±156 | 1 | 2 | 0 | 7 |
| LIB | 10 | 555±178 | 1 | 1 | 1 | 7 |
| GEM | 10 | 301±134 | 1 | 4 | 1 | 4 |
| LIB-GEMs | 10 | 345±163 | 1 | 3 | 2 | 4 |
| PTK7-GEMs | 10 | 156±78 | 2 | 6 | 2 | 0 |

| **Table S6.** Hematological and biochemical data from sacrificed mice. | | | | | | |
| --- | --- | --- | --- | --- | --- | --- |
| Name | PBS | LIB | PTK7 | GEM | LIB-GEMs | PTK7-GEMs |
| WBC (10^9^/L) | 4.5 ± 0.9 | 4.7 ± 1.8 | 4.7 ± 1.7 | 4.9 ± 1.2 | 4.6 ± 1.1 | 3.8 ± 1.3 |
| RBC (10^12^/L) | 8.9 ± 0.3 | 8.8 ±0.2 | 8.8 ± 0.3 | 8.4 ± 0.3 | 8.5 ± 0.8 | 8.4 ± 0.6 |
| PLT (10^9^/L) | 761.7 ± 59.7 | 756.6 ± 87.3 | 747.6 ± 340.2 | 752.0 ± 101.7 | 764.5 ± 94.3 | 720.6 ± 95.4 |
| HGB(g/L) | 150 ± 4.0 | 151 ± 3.3 | 148.8 ± 6.4 | 145 ± 4.6 | 141.5 ± 12.4 | 143.8 ± 15.2 |
| ALT(U/L) | 33.9 ± 3.4 | 39.0 ± 4.9 | 39.6 ± 4.7 | 48.2 ±7.5* | 46.7 ± 4.7* | 38.0 ± 2.7 |
| AST(U/L) | 196.9 ± 23.2 | 186.4 ± 40.2 | 208.6 ± 43.3 | 248.2 ± 38.3* | 242.7 ± 17.2* | 174.1 ± 19.4 |
| CK(U/L) | 1224.7 ± 143.8 | 1321.5 ± 228.4 | 1287.1 ± 451.8 | 1321.7 ± 142.7 | 1338.0 ± 138.9 | 1350.9 ± 146.5 |
| CREA (μmol L^-1^) | 18.1 ± 3.0 | 19.8 ± 4.5 | 18.8 ± 3.9 | 18.1 ± 1.5 | 17.7 ± 5.4 | 16.8 ± 5.0 |
| UREA (mmol L^-1^) | 8.4 ± 1.2 | 8.0 ± 1.0 | 8.9 ± 2.7 | 8.7 ± 0.4 | 8.6 ± 1.6 | 7.7 ± 1.0 |
| White blood cells (WBC), red blood cells (RBC), platelets (PLT), hemoglobin (HGB), alanine aminotransferase (ALT), aspartate aminotransferase (AST), creatine phosphokinase (CK), creatinine (CR), and urea Data were mean ± SEM, n=5; **P* < 0.05 vs. PBS group and PTK7-GEMs group. | | | | | | |
